# MSK1 is required for the beneficial synaptic and cognitive effects of enriched experience across the lifespan

**DOI:** 10.18632/aging.204833

**Published:** 2023-07-10

**Authors:** Lorenzo Morè, Lucia Privitera, Daniel D. Cooper, Marianthi Tsogka, J. Simon C. Arthur, Bruno G. Frenguelli

**Affiliations:** 1 School of Life Sciences, University of Warwick, Coventry CV4 7AL, UK; 2 School of Pharmacy and Biomedical Sciences, University of Central Lancashire, Preston PR1 2HE, UK; 3 School of Life Sciences, University of Dundee, Dundee DD1 5EH, UK

**Keywords:** cognitive reserve, synaptic plasticity, anxiety, spatial memory, LTP

## Abstract

Positive experiences, such as social interaction, cognitive training and physical exercise, have been shown to ameliorate some of the harms to cognition associated with ageing. Animal models of positive interventions, commonly known as environmental enrichment, strongly influence neuronal morphology and synaptic function and enhance cognitive performance. While the profound structural and functional benefits of enrichment have been appreciated for decades, little is known as to how the environment influences neurons to respond and adapt to these positive sensory experiences. We show that adult and aged male wild-type mice that underwent a 10-week environmental enrichment protocol demonstrated improved performance in a variety of behavioural tasks, including those testing spatial working and spatial reference memory, and an enhancement in hippocampal LTP. Aged animals in particular benefitted from enrichment, performing spatial memory tasks at levels similar to healthy adult mice. Many of these benefits, including in gene expression, were absent in mice with a mutation in an enzyme, MSK1, which is activated by BDNF, a growth factor implicated in rodent and human cognition. We conclude that enrichment is beneficial across the lifespan and that MSK1 is required for the full extent of these experience-induced improvements of cognitive abilities, synaptic plasticity and gene expression.

## INTRODUCTION

The increased longevity associated with improved health care, sanitation and nutrition has resulted in larger numbers of people living with the consequences of age-related cognitive decline and dementias. Such conditions impose heavy burdens on the elderly, their careers, health-care providers and society in general [1, 2]. Attempts to mitigate the impact of ageing on the brain have included non-pharmacological interventions, such as social interaction, cognitive stimulation and physical exercise [3–9], in effect, essentially enriching the day-to-day experiences of the elderly.

That experience exerts a profound influence on the structure and function of the mammalian brain has been known for many years [10]. A convenient means to study the influence of experience on the nervous system is to expose experimental animals to environmental enrichment, a manipulation that provides animals with a larger social group, a higher level of novelty, greater opportunities for exploration, and to increased physical exercise. Initially conceived by Donald Hebb to demonstrate that genetics alone did not determine intelligence [11, 12], this protocol induces neurogenesis, greater dendritic spine density, enhanced synaptic plasticity, profound changes in gene expression, and ultimately improved performance across a range of behavioural tasks [13–18]. These benefits are observed across the lifespan, including into old age [3, 19–25] and in experimental models of age-related and other neurodegenerative and neurological disorders [26–31]. While providing models and parallels for similar enrichment strategies in the elderly human, such studies have additionally prompted attempts to harness the beneficial effects of enrichment through pharmacological “enviromimetics” to halt or even reverse age-related cognitive decline [14, 32, 33].

Recently, a neuronal protein kinase, mitogen- and stress-activated protein kinase 1 (MSK1) has been identified as being a prime effector within the mammalian brain of the beneficial effects of enrichment in the early phase of the lifespan (birth to 4 months) [34–38]. MSK1 plays a pivotal role in transducing the additional sensory experiences associated with enrichment into long-lasting changes in gene expression, neuronal morphology and synaptic activity, and ultimately to improvements in cognition [16]. MSK1 is activated by brain-derived neurotrophic factor (BDNF) and regulates the expression, notably via the phosphorylation of CREB and histone H3 [39, 40], of a range of genes including those for the key plasticity-related proteins Arc/Arg3.1 and EGR1 [36, 41]. Importantly, BDNF has been implicated in human cognition, hippocampal volume increases, and the response to physical exercise [42–44]. Furthermore, MSK1 is expressed in neurons of the hippocampus [38, 45–48], a major brain structure that underpins certain forms of learning and memory, which is shaped by experience [49], benefits from environmental enrichment [16, 50], and is particularly affected by normal ageing and neurodegenerative dementias such as Alzheimer’s disease [51].

Using mice harbouring a knock-in point mutation of the MSK1 gene, which results in the elimination of the kinase activity of MSK1 (kinase dead; MSK1 KD), we previously showed that the kinase activity of MSK1 was required for homeostatic synaptic scaling *in vitro*, and the *in vivo* enrichment-induced enhancement of miniature excitatory postsynaptic currents [34, 37]. More recently we have found that the kinase activity of MSK1 is necessary for the full benefits of enrichment on cognition, in particular, in the strength of hippocampal spatial memory and cognitive flexibility. This may be due to an experience- and MSK1-dependent expansion of the synaptic dynamic range as evidenced by greater LTP and LTD in enriched wild-type (WT), but not MSK1 KD mice [36]. Via an RNA-Seq analysis of the hippocampal transcriptome under standard and enriched conditions, we also showed a crucial role for MSK1 in the experience-dependent regulation of gene expression, and the initiation of a genomic homeostasis characterised by the unexpected downregulation of key plasticity-related proteins such as EGR1 and Arc/Arg3.1 [16, 36].

These findings, while providing novel insight into the mechanistic basis of the beneficial effects of enrichment, did not address the role of MSK1 throughout the lifespan, and especially during the latter period of life when cognitive decline is more prevalent and pressing, and for which effective therapies to halt or reverse this decline are lacking. To this end we have studied the role of MSK1 on cognitive performance, synaptic plasticity and dendritic spine density in healthy mice that underwent an extensive 10-week environmental enrichment protocol during two distinct periods of life. One group underwent enrichment starting at 6 months of age, with testing starting at 8.5 months of age (Adult mice). A second group underwent the same protocol starting at 18 months of age and with testing initiated at 20.5 months of age (Aged mice), and in which we additionally conducted an RNA-Seq analysis of experience- and MSK1-dependent hippocampal gene expression.

We provide experimental evidence that a 10-week environmental enrichment protocol, which had some benefits in Adult mice, was sufficient to improve spatial memory in WT, but not MSK1 KD Aged mice. Moreover, these benefits of enrichment were associated with enhancements of LTP at both ages, but largely absent in mice lacking the kinase activity of MSK1. The benefits of enrichment in WT Aged mice compared to MSK1 KD mice likely reside in the MSK1-dependent up-regulation of hippocampal gene expression observed in enriched WT mice. We propose that MSK1 is necessary for the brain to embody the full molecular and cellular benefits associated with enriched experience, and which lead to the remediation of age-related cognitive decline.

## RESULTS

### Enrichment influences motor and anxiety-like behaviours in an age- and MSK1-dependent manner

**Figure 1:**
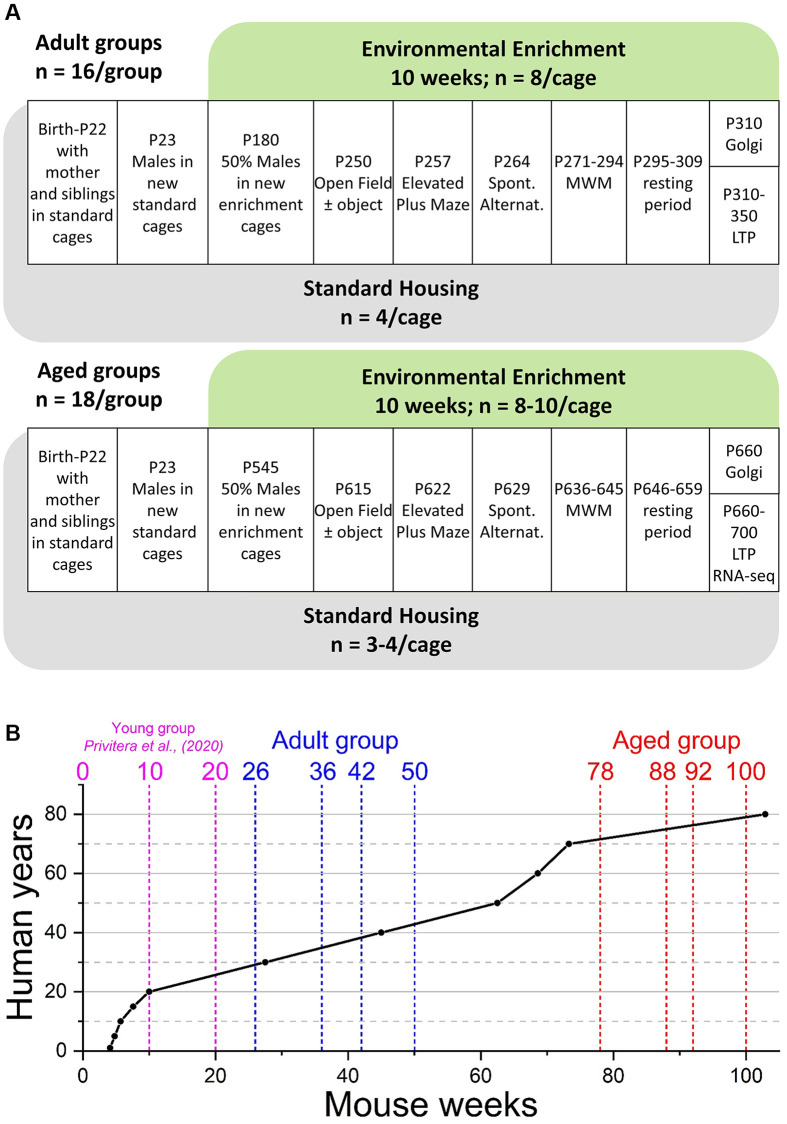
**Experimental outline and age comparison.** (**A**) Time-course of housing provision and experimental treatments for Adult mice (upper panel) and Aged mice (lower panel). Abbreviations: P: postnatal day; MWM: Morris water maze; LTP: long-term potentiation and electrophysiological analysis of synaptic transmission. (**B**) Comparison of mouse and human ages based on data from Wang et al., (2020) [71]. The broken vertical lines represent the weeks at which two separate cohorts of mice (blue; Adult and red; Aged): went into enrichment (weeks 26 and 78); behavioural testing started (weeks 36 and 88), behavioural testing ended (weeks 42 and 92), and the end of *ex vivo* analyses (weeks 50 and 100). For comparison, the previously reported young group (cyan) were born into standard housing or enrichment (week 0), began behavioural testing at week 10, with all testing completed by week 20 [36].

Since the main aim of the behavioural testing was to assess higher cognitive functions, such as hippocampus-dependent spatial working and reference memory (Figure 1A), we adopted a bottom-up approach to identify potential confounding factors due to physiological, neurological and emotional influences on behaviour that might be occasioned by housing conditions, genotype or age, and which might have had an impact on cognition [52]. We first assessed the general health of all the mice using a standardized neurological test well adapted to mice [36, 53] to reveal any gross motor impairments that could be expected in aged mice. No animals in any of the Adult or Aged (Figure 1B) groups showed any neurological signs (data not shown). The neurological health of all animals in this study thus avoided potential motor confounds that could influence performance on any of the behavioural tests.

**Figure 2:**
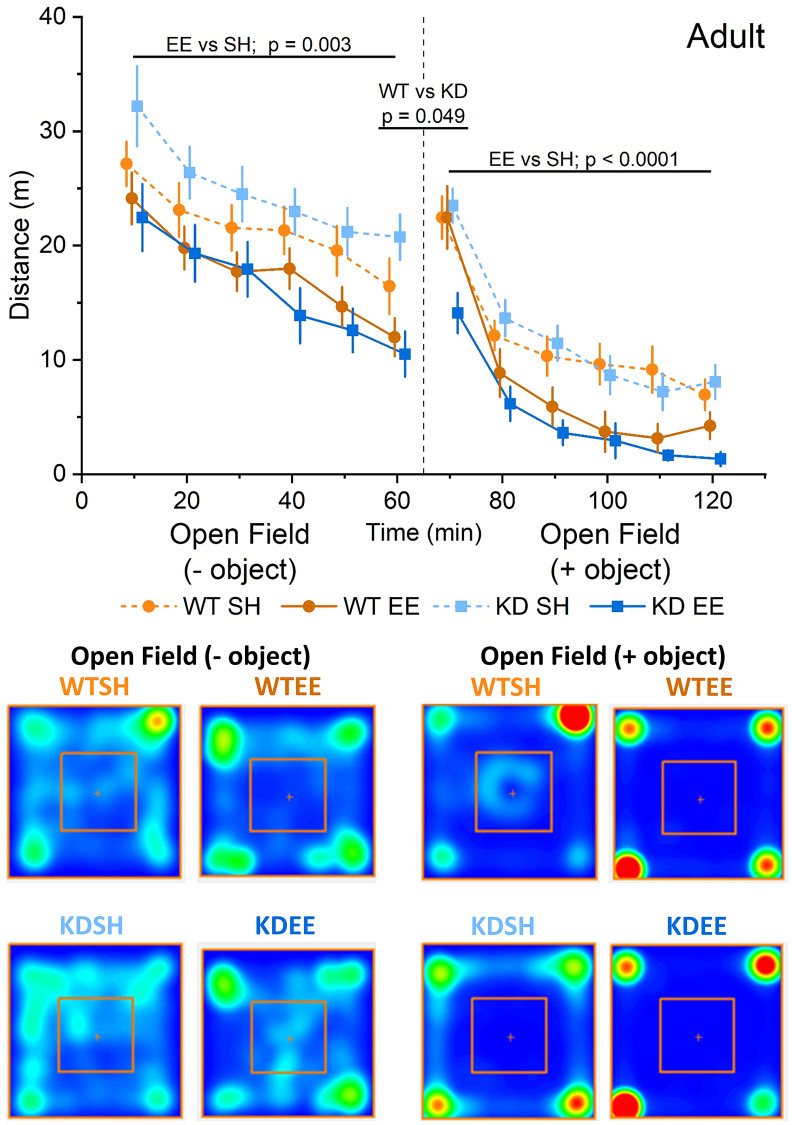
**Enrichment reduces novelty-induced locomotion in adult mice.** An RM-ANOVA on the open field task in the Adult groups showed an effect of Time for all groups (F(5,300) = 49.74, *p* < 0.0001) indicating a reduction in activity over time, and also a Housing effect (F(1,60) = 9.88, *p* = 0.003) showing that mice in the enriched groups were less active regardless of their genotype. Following the introduction of an object into the arena (broken vertical line; 50 ml plastic Falcon tube), exploration increased from the previous level of activity at 60 mins. There was a borderline genotype effect (F(1,60) = 4.02, *p* = 0.049) with the WT group showing an enhanced response to novelty. Subsequently, enrichment reduced exploration in both genotypes following the introduction of an object, with a significant interaction of Time x Genotype x Housing (F(5,300) = 51.75 *p* = 0.012). In this open field + object stage an RM-ANOVA also showed an effect of Time for all groups (F(5,300) = 128.53, *p* < 0.0001) indicating a reduction in activity over time, and also a Housing effect (F(1,60) = 17.03, *p* < 0.0001) showing again that enriched mice were less active in response to the introduction of an object regardless of their genotype. The data are presented as distance travelled in metres as cumulative distance reported in 10-min intervals. 60 min per stage (120 min in total). Datapoints are presented as mean ± SEM. The heatmaps below the graphs depict the arena occupancy in the open field ± object stages for the mice of each group.

To confirm that our enrichment protocol had tangible effects on animal behaviour, we initially assessed the influence of enrichment on open field and novelty-induced locomotion, and anxiety-like behaviour, as both are sensitive to enrichment [36, 54]. WT and MSK1 KD Adult mice raised from birth in standard housing behaved similarly when exposed to an open field arena, in that activity declined over time (Figure 2). However, in contrast to their counterparts raised in standard housing, Adult enriched animals of both genotypes displayed reduced locomotor activity in the open field throughout the entire period of open field exploration, as previously reported for young mice [36], and suggestive of prior habituation to larger and novel environments. The introduction of an object into the centre of the open field as a novelty stimulus provoked greater exploration in standard-housed and enriched WT mice, compared to the preceding level of activity at 60 min. In contrast, the response in MSK1 KD mice to novelty was significantly blunted, despite similar amounts of time spent facing the object across the four groups (Supplementary Figure 1A). After this initial difference, Adult enriched mice of both genotypes subsequently showed reduced exploration of the open field arena compared to their standard-housed counterparts.

**Figure 3:**
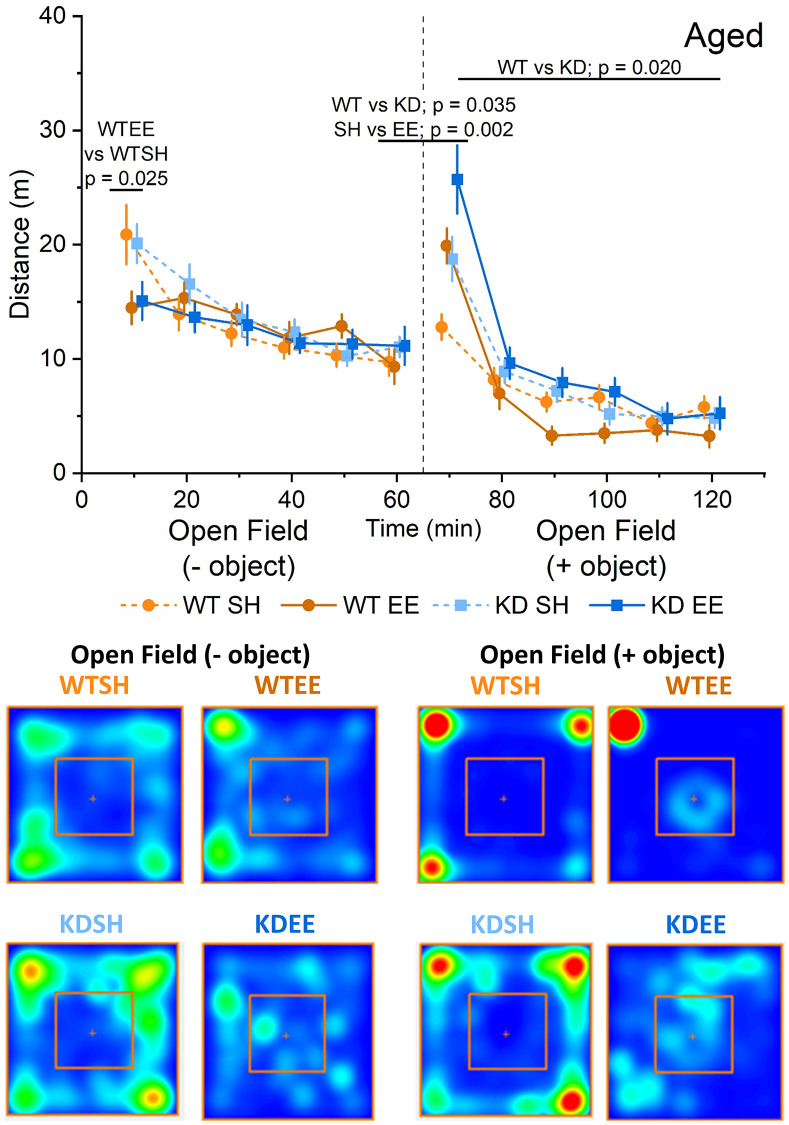
**Enrichment reduces novelty-induced locomotion in aged mice.** In the Aged groups an RM-ANOVA showed an effect of Time for all groups on the distance travelled (F(5,260) = 27.53, *p* < 0.0001) indicating a reduction in activity over time. There was also a Time x Housing effect (F(52,260) = 6.25, *p* < 0.0001), but the Simple Main Effects showed this was significant only for the first 10 minutes of the OF between WTSH and WTEE (*p* = 0.025), but not between KDSH and KDEE (*p* = 0.077). Following the introduction of an object (broken vertical line) an analysis of the novelty-induced locomotor shift was carried out comparing levels of activity during the first 10 minutes from the introduction of the object with the last 10 minutes of the open field. A significant effect for both Genotype and Housing was found (F(1,52) = 4.71, *p* = 0.035 and F(1,52) = 10.34, *p* = 0.002, respectively) indicating a greater effect of novelty in the enriched animals and in the MSK1 KD mutants. In the open field + object phase of the trial, an RM-ANOVA showed an effect of Time for all groups (F(5,260) = 132.43, *p* < 0.0001) reflecting a reduction in activity over time. There was a Time x Housing and a Time x Genotype effect (F(52,260) = 10.43, *p* < 0.0001 and F(52,260) = 4.20, *p* = 0.001, respectively) and also a Genotype effect; F(1,52) = 5.78, *p* = 0.020 indicating a greater activity in the MSK1 KD mutant mice, and with lower levels of activity in the enriched WT mice. The data are presented as distance travelled in metres as cumulative distance reported in 10 min intervals. Each phase lasted 60 min (120 min in total). Datapoints are presented as mean ± SEM. Heatmaps below the graphs depict the arena occupancy in the open field ± object stages for the mice of each group.

Aged mice showed a different pattern of behaviour in the open field with similar levels of exploration across genotypes and housing condition, except for the first 10 minutes when standard-housed mice showed greater exploration compared to enriched mice (Figure 3). In response to the introduction of the object, where again all groups faced the object for comparable periods of time (Supplementary Figure 1B), all groups except the standard-housed WT mice showed increased exploration. Thereafter, only the enriched WT group showing reduced exploratory behaviour, as per the Adult mice, and in contrast to enriched Aged MSK1 KD mice, which behaved similarly to the standard-housed mice of both genotypes.

**Figure 4:**
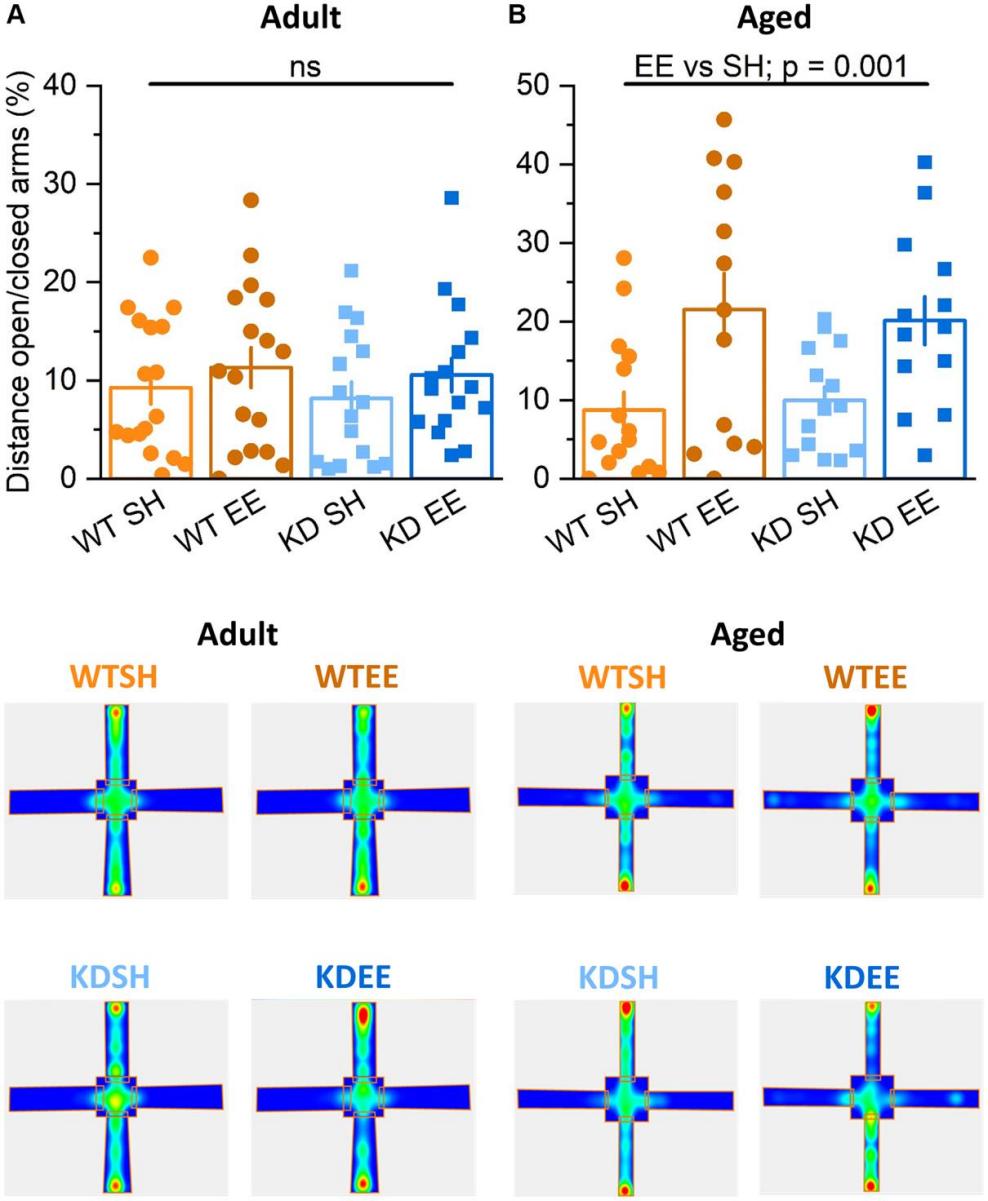
**Enrichment reduces anxiety-like behaviour in aged mice.** (**A**) In the Elevated Plus Maze task in the Adult groups the distance travelled in the open area vs. that in the closed area (Distance travelled in the open arms/distance travelled in the closed arms ×100 expressed in %.) was not different between Genotype nor Housing condition (Genotype effect: F(1,56) = 0.12, *p* = 0.740; Housing effect: F(1,56) = 3.87, *p* = 0.054; Interaction Genotype x Housing: F(1,56) = 0.81, *p* = 0.370). (**B**) In the Aged groups the distance travelled in the open area vs. that in the closed area was greater in the enriched groups of both genotypes, indicating that enrichment effectively reduced anxiety-like behaviour (Housing: F(1,49) = 13.81, *p* = 0.001). Heatmaps below the graphs depict the arm occupancy for the mice of each group, with the horizontal arms being the open arms in all conditions. Individual data points are presented for each animal, with the bar graph representing the mean ± SEM of the data.

In the elevated plus maze test for anxiety-like behaviour, there was no influence of genotype or housing on the behaviour of Adult mice in terms of distance travelled (Figure 4A) or time spent (Supplementary Figure 1C) in the open arms. In contrast, Aged mice of both genotypes raised in the enriched environment travelled further (Figure 4B) and spent more time (Supplementary Figure 1D) in the open arms of the elevated plus maze. This likely reflects reduced anxiety-like behaviour, a classic effect of environmental enrichment [55], and was also seen in a previous study of younger enriched WT and MSK1 KD mice [36]. These observations confirm the effectiveness of the enrichment protocol in influencing behaviour in both Adult and Aged WT and MSK1 mutant mice, but reveal age- and experience-dependent differences on locomotion (Adult > Aged) and anxiety-like behaviour (Aged > Adult).

### MSK1 is necessary for preserving spatial working memory and spatial reference memory in aged mice

**Figure 5:**
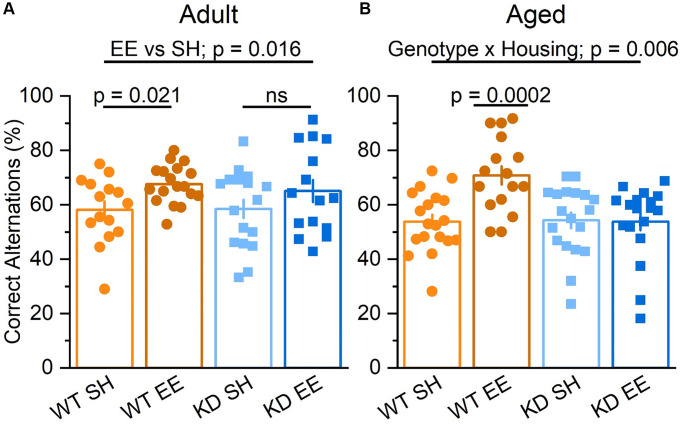
**Enrichment improves hippocampus-dependent spatial working memory via MSK1.** (**A**) In the Adult groups the percentage of correct alternations was higher for mice in enrichment (Housing: F(1,61) = 6.10 *p* = 0.016). Although there was no significant interaction, the difference between EE and SH was greater in the WT mice, which showed a significant improvement (F(1,61) = 5.64 *p* = 0.021) compared to the MSK1 KD which showed no significant (ns) improvement in performance compared to their standard-housed counterparts (F(1,61) = 1.27 *p* = 0.270). (**B**) In the Aged groups there was a significant interaction of Genotype x Housing F(1,68) = 8.17 *p* = 0.006. The Simple Main Effect analysis showed that the difference between EE and SH was greater in the WT which showed a highly significant improvement F(1,68) = 15.04 *p* = 0.0002 compared to the KD which showed none F(1,68) = 0.01 *p* = 0.920. Results are expressed as arm entries sequence over 10 minutes for which a correct alternation % was calculated with the criterion of one repetition across 5 entries. These results indicate that the benefit of enrichment was exclusive to WT. Individual data points are presented for each animal, with the bar graph representing the mean ± SEM of the data.

To assess hippocampus-dependent forms of spatial working memory we first employed a spontaneous alternation task, which has been shown to be a reliable tool to assess mutant mice with neurological conditions [56, 57]. While Adult standard-housed mice of both genotypes performed at comparable levels, there was a significant effect of enrichment, with enriched mice making more correct alternations, an effect that was statistically more pronounced in enriched WT mice (Figure 5A). In the Aged mice, the influence of genotype and housing on spatial working memory was even more stark, with a clear genotype x housing interaction in which the benefits of enrichment were only observed in the enriched WT mice (Figure 5B). These data suggest that the benefits of enrichment on spatial working memory becoming increasingly dependent on MSK1 as ageing progresses.

To investigate a more cognitively demanding form of hippocampus-dependent spatial memory, we used the Morris water maze task. To allow for a direct comparison across the ages of mice, we accounted for the reduced swimming speed of the Aged groups when compared to younger mice (Supplementary Figure 2A, 2B) by analysing the distance swum, rather than the time taken to reach the escape platform. We also limited trial exposure to 90 s, as opposed to the usual 120 s [36], to avoid the possibility of fatigue or hypothermia in Aged mice. As a further control for the sensorimotor abilities in the Adult and Aged mice we also ran a visual test before starting the learning stage to assess the ability of mice to see visual cues, a basic function upon which spatial memory relies, and their motivation to escape from immersion. This, together with the previous habituation stage, ensured that all mice were proficient swimmers and had experience in swimming and navigating to a platform before the learning session started. Accordingly, all mice of both age groups performed proficiently and similarly on the visual cue task, with no differences between the groups (data not shown). The absence of performance biases across genotypes, housing, and ages thus precluded confounds associated with different levels of sensorimotor ability, anxiety, motivation or fatigue.

**Figure 6:**
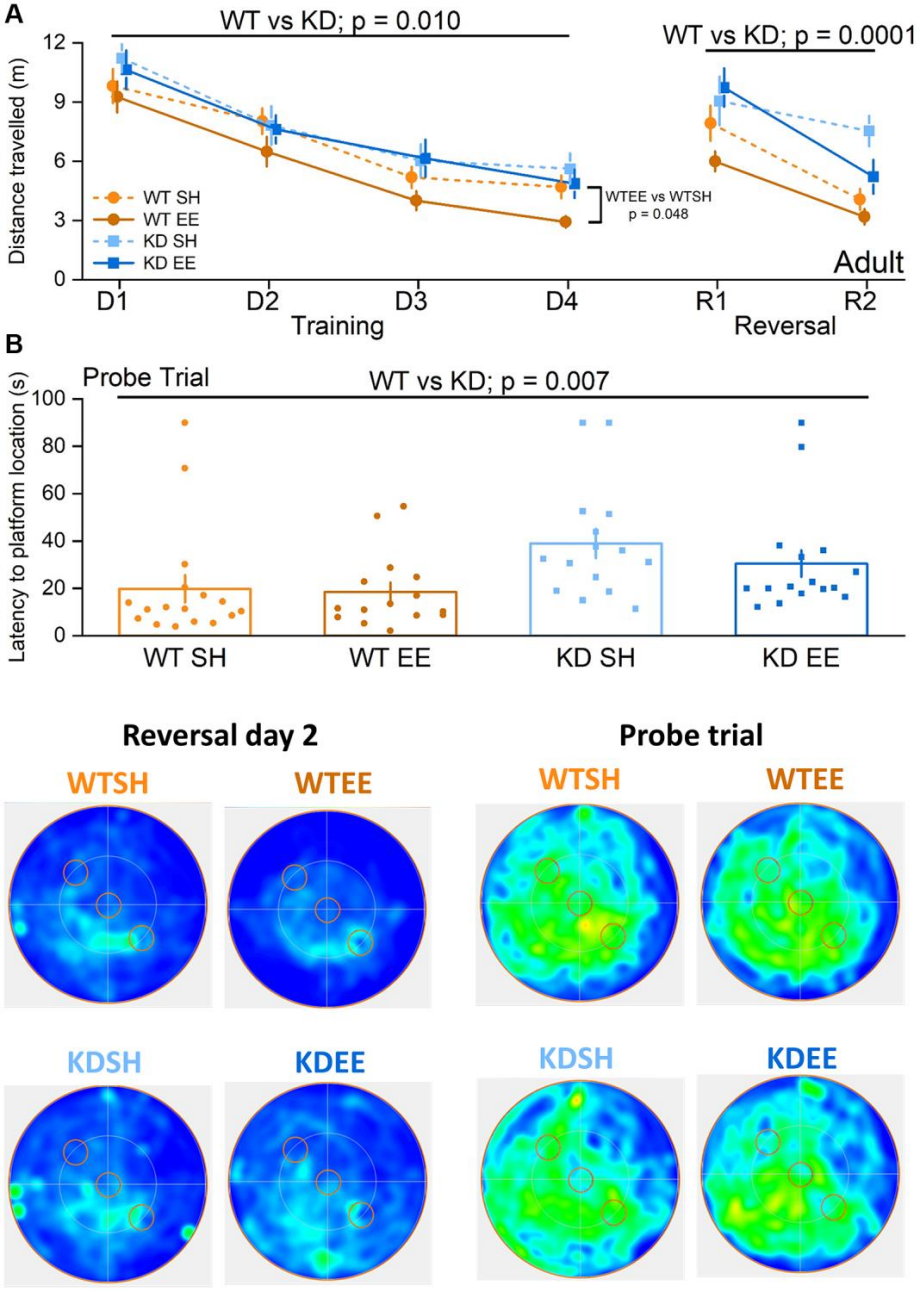
**Environmental enrichment improves cognitive flexibility in adult mice via MSK1.** No differences were observed across groups in the visual cue version of the test (Genotype: F(1,59) = 0.09, *p* = 0.760; Housing: F(1,59) = 1.17, *p* = 0.280; Genotype x Housing: F(1,59) = 2.57, *p* = 0.110; data not shown) suggesting comparable levels of visual acuity, swimming ability and motivation to navigate to the platform. (**A**) All four Adult groups showed learning over the first stage of training (Days (D) 1–4). The RM-ANOVA on distance swum to reach the escape platform showed a significant effect of Session F(3,177) = 52.38, *p* < 0.0001. It also showed a main effect of Genotype F(1,59) = 7.19 *p* = 0.010 where WT mice performed better than MSK1 KD mutant mice. Although there was no significant interaction of Genotype x Housing, the enriched WT mice were significantly better than standard-housed WT mice (F(1,59) = 4.09 *p* = 0.048) while no significant difference was seen between standard-housed or enriched MSK1 KD mice (F(1,59) = 0.32, *p* = 0.570). On the Reversal (R) learning stage (2 days) the RM-ANOVA on the distance swum to reach the new escape platform location showed an effect of session F(1,59) = 36.53 *p* < 0.0001 and Genotype F(1,59) = 16.97, *p* = 0.0001 indicating that all groups learned over the time but WT mice performed better than MSK1 KD mice. Although there was no significant interaction, the WT mice performed much better than the MSK1 KD mice in both standard and enriched housing (F(1,59) = 6.77, *p* = 0.012 and F(1,59) = 10.37, *p* = 0.002, respectively). Data are presented as mean ± SEM. (**B**) On the probe trial 24 hours later WT mice showed the best goal directed behaviour for the reversed location of the escape platform (south west quadrant) and the standard-housed MSK1 KD mutant mice showed the worst performance. The Univariate ANOVA on the total time spent in the reversal quadrant (where the platform had been last) showed a strong effect for Genotype (F(1,59) = 9.15, *p* = 0.004; not shown). The effect of genotype was also significant for latency to enter the Reversal platform area (F = 7.91 *p* = 0.007). Although the interaction did not reach significance, the Simple Main effects showed a significant difference on the time spent in the reversal platform quadrant between enriched WT mice and enriched MSK1 KD mice (F(1,59) = 9.28, *p* = 0.003) and between standard-housed WT and MSK1 KD mice on the latency to reach the reversal platform location (F(1,59) = 6.07, *p* = 0.017). Heatmaps below the graphs depict the arena occupancy for the mice of each group during the last day of reversal learning and the Probe trial. Individual data points are presented for each animal, with the bar graph representing the mean ± SEM of the data.

In the Adult groups during the learning (Training) phase of the water maze the WT mice performed better than the MSK1 KD mice (Figure 6A). While there was no genotype x housing interaction, enriched WT mice performed significantly better than standard-housed WT mice, whereas no such differential was observed between the MSK1 mutant mice. Similarly, when a Reversal learning stage was carried out after the learning phase, WT mice performed better than the MSK1 KD mutant mice (Figure 6A). A more detailed between factors statistical analysis showed that, while there was no significant genotype x housing interaction, the difference between enriched WT and MSK1 KD mice was highly significant indicating that enrichment benefited WT mice more than the MSK1 KD mutants in this test of cognitive flexibility. Measurements of the latency to the platform location during the Training and Reversal phases were consistent with those obtained with distance travelled (Supplementary Figure 2C). A similar genotype effect, where the WT outperformed the MSK1 KD mice, was also seen in the Probe trial for memory persistence administered 24 hrs later (Figure 6B).

**Figure 7:**
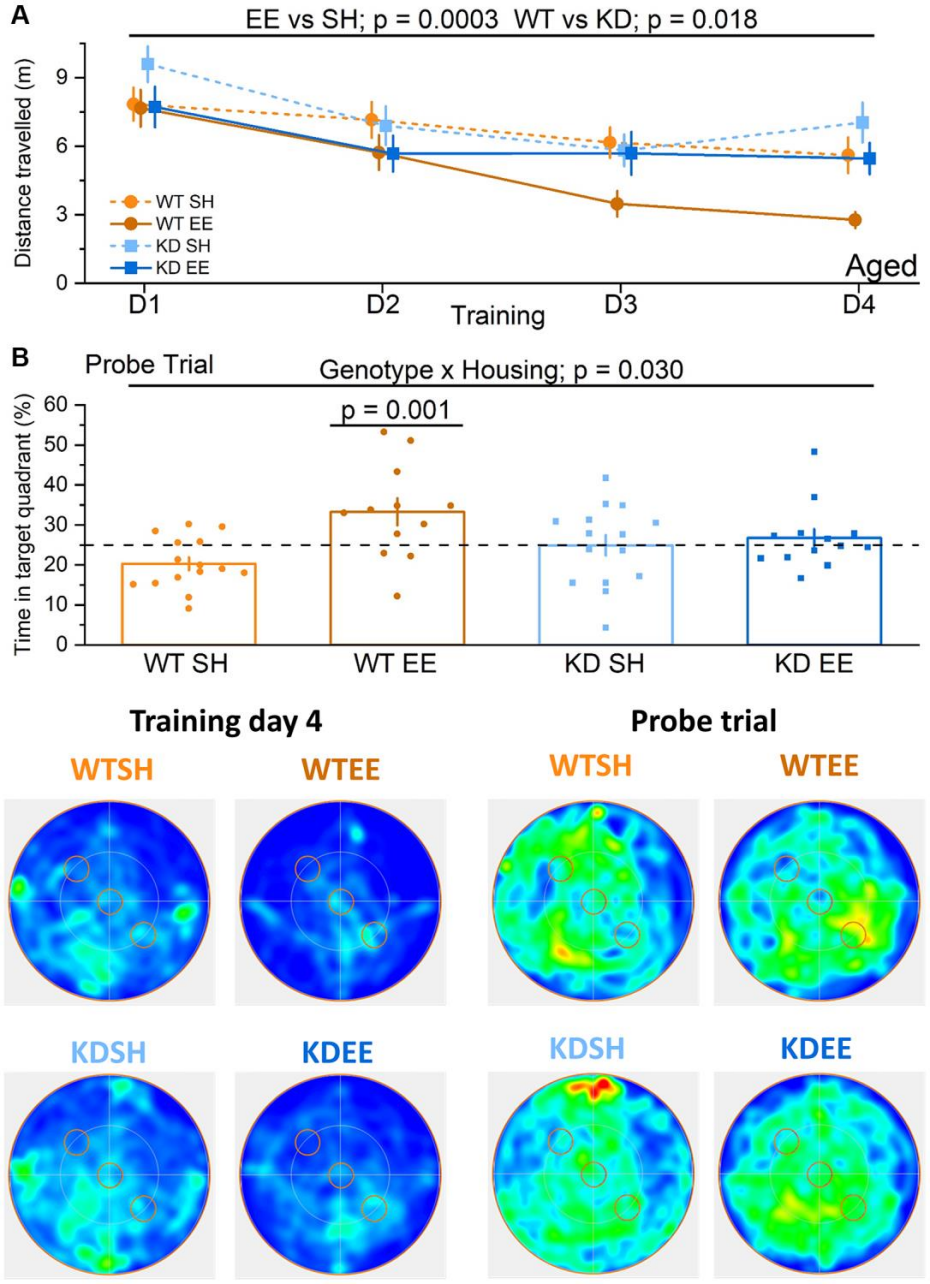
**Enrichment of aged mice improves hippocampus-dependent reference memory via MSK1.** No differences were observed across groups in the visual cue version of the test (Genotype: F(1,51) = 0.001, *p* = 0.970; Housing: F(1,51) = 0.37, *p* = 0.570; Genotype x Housing F(1,51) = 2.26, *p* = 0.140; data not shown) suggesting comparable levels of visual acuity, swimming ability and motivation to navigate to the platform. (**A**) In the Aged groups the enriched WT mice were the best performers and did not show the age-related impairment seen in standard-housed WT mice. The RM-ANOVA analysis on the distance travelled to reach the escape platform showed a significant effect of Session F(3,153) = 12.63, *p* < 0.0001. Importantly the standard-housed WT mice did not show any significant learning improvement in this period F(3,42) = 1.95, *p* = 0.136. The analysis also showed a main effect of the Housing (F(1,51) = 15.23 *p* = 0.0003) where enriched mice performed better than standard-housed mice, and of Genotype (F(1,51) = 6.00 *p* = 0.018) where WT mice performed better than MSK1 KD mice. Although there was no significant interaction, on training day 3 and 4 the WT enriched mice clearly outperformed all the other groups. Data are presented as mean ± SEM. (**B**) On the probe trial the enriched WT mice had the best performance. The ANOVA showed a significant effect of Housing (F(1,51) = 8.84, *p* = 0.004), indicating a better performance of enriched mice, but the significant Genotype x Housing interaction (F(1,51) = 4.98, *p* = 0.030) and the Simple Main effects analysis showed that the difference between standard-housed mice and enriched mice was only significant in the WT mice (F(1,51) = 13.26, *p* = 0.001). Heatmaps below the graphs depict the arena occupancy for the mice of each group during the last day of training and the Probe trial. Individual data points are presented for each animal, with the bar graph representing the mean ± SEM of the data. Abbreviation: ns: not significant.

In the Aged groups, the benefits of enrichment were selectively observed in WT mice even during the Training stage (Figure 7A), when their performance level was comparable to that of the younger enriched WT Adults (Figure 6A). In contrast, there was little evidence of robust learning in standard-housed WT or MSK1 KD mice, or indeed in enriched MSK1 KD mice (Figure 7A). Similar observations were made with measurements of latency to platform location (Supplementary Figure 2D). The paucity of learning in these latter three groups obviated conducting the Reversal learning stage since the location of the initial target platform had not been well consolidated. The clear benefits of enrichment in Aged WT mice was also evident in the Probe trial where a post-hoc analysis after a significant genotype x housing interaction showed that enriched WT mice spent more time in the Training quadrant, compared to the other groups (Figure 7B). These data indicate that the full cognitive benefits of enrichment in the acquisition of spatial locations, reversal learning of the task, and persistence of the memory depend upon MSK1 in an increasingly age-dependent manner.

### MSK1 underpins the experience-dependent enhancement of synaptic plasticity across the lifespan

**Figure 8:**
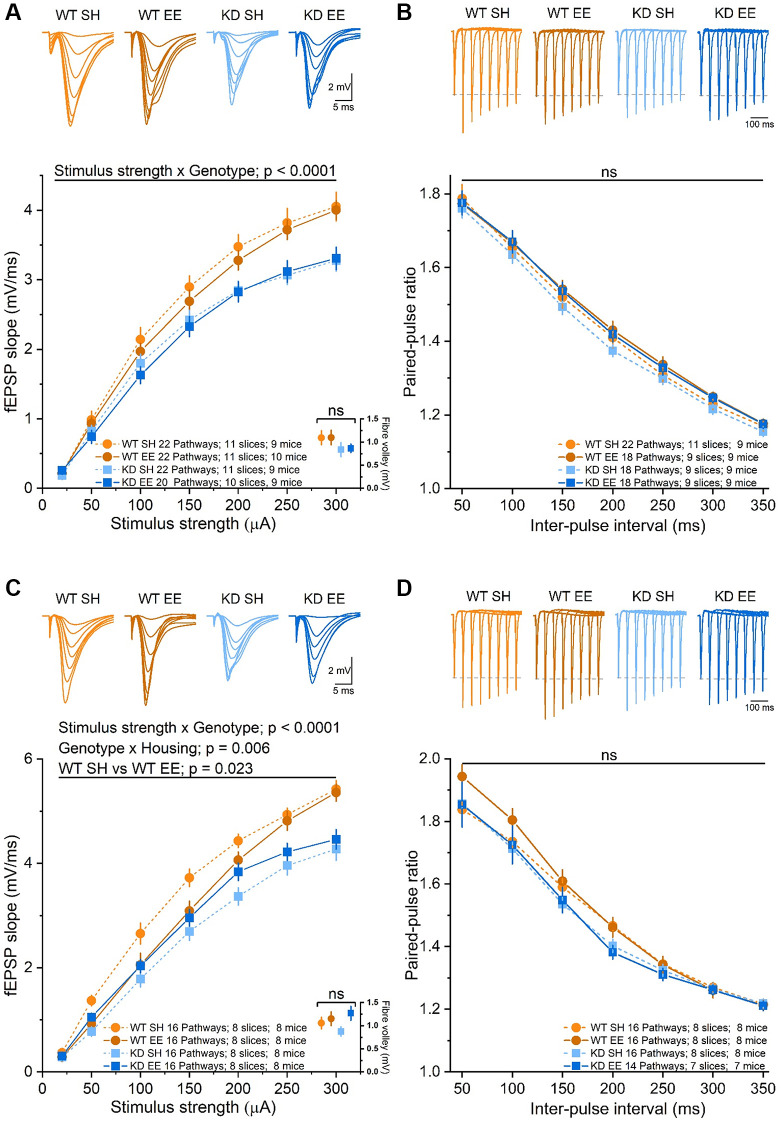
**Basal synaptic transmission of stratum radiatum in area CA1 is affected in an experience- and MSK1-dependent manner.** (**A**) The RM-ANOVA on the fEPSP Input/Output profile in the Adult groups with a within-factor as stimulus strength (20 to 300 μA) gave a significant effect (F(6,492) = 958.85, *p* < 0.0001) and an interaction of stimulus strength x Genotype: F(6,492) = 10.09, *p* < 0.0001 but no significant interaction of stimulus strength x Housing, nor stimulus strength x Genotype x Housing. There was also a significant effect of Genotype (F(1,82) = 13.07, *p* = 0.001), but no significant interaction Genotype x Housing nor Housing alone. A comparison of the fibre volley amplitude across a subset of experiments (10–12 pathways from 7–9 slices and from 7–9 mice) where the fiber volley was measurable at 300 μA (inset, bottom right, data points offset for clarity) showed no significant (ns) effect of Genotype, Housing, or Genotype x Housing across groups. (**B**) The RM-ANOVA on the paired-pulse facilitation in the Adult groups with inter-pulse interval (50–350 ms) as a within-factor variable gave only a significant effect of interval (F(6,432) = 1229.1, *p* < 0.0001) but not of Genotype or Housing. (**C**) In the Aged groups there was a significant effect of stimulus strength (F(6,360) = 787.75, *p* < 0.0001) and an interaction between stimulus strength x genotype (F(6,360) = 7.18, *p* < 0.0001). The between effects analysis revealed a significant effect of genotype (F(1,60) = 28.22, *p* < 0.0001) and an interaction Genotype x Housing (F(1,60) = 8.26, *p* = 0.006). The simple main effect analysis revealed a significant difference between standard-housed and enriched WT mice (F(1,60) = 5.44, *p* = 0.023) and between standard-housed WT and MSK1 KD mice (F(1,60) = 33.51, *p* < 0.0001). A comparison of the fibre volley amplitude across a subset of experiments (10–14 pathways from 6–9 slices and from 6–9 mice) showed no significant (ns) effect of Genotype, Housing, or Genotype x Housing across groups. (**D**) In the analysis of the paired-pulse facilitation data, there was only a significant effect of interval (F(6,348) = 614.28, *p* < 0.0001), but not of Genotype or Housing. Data are presented as mean ± SEM.

Environmental enrichment has repeatedly been shown to influence synaptic activity [15, 16, 50]. To establish the extent to which changes in synaptic function could underlie the age-, enrichment- and MSK1-dependent effects on spatial working and reference memory, we conducted dual-pathway extracellular recordings from area CA1 of hippocampal slices prepared from the eight groups of experimental animals (2 × genotype, 2 × housing, 2 × age), as previously described [36, 58]. In Adult mice we replicated the observations made previously [36, 58] of a deficit in basal synaptic transmission in the MSK1 KD mice (Figure 8A). This was not associated with an effect of the mutation on axon excitability, as measured by the amplitude of the presynaptic fibre volley, that could account for the differences in basal synaptic transmission (Figure 8A). Similarly, there was no effect on paired-pulse facilitation (PPF; Figure 8B), indicative of similar probabilities of glutamate release between the two genotypes. While these parameters were not obviously affected by enrichment, there was a tendency for the fEPSP to be smaller in enriched WT mice, and for PPF to be increased, but with no change in the presynaptic fibre volley, as we observed in younger animals [36]. These observations are internally consistent as a decrease in the probability of neurotransmitter release would be reflected in both increased PPF and smaller fEPSPs.

In Aged mice, where there was no influence of housing or genotype on the presynaptic fibre volley, enrichment had a significant depressant effect of the fEPSP in WT mice (Figure 8C), with evidence of enhanced PPF at shorter inter-pulse intervals (Figure 8D). In contrast, in enriched Aged MSK1 KD mice there was a marginal increase in the fEPSP (Figure 8C), but without affecting PPF (Figure 8D). Overall, these observations, consistent with those made previously in younger animals [36], suggest that enrichment reduces synaptic transmission in WT mice in an age- and MSK1-dependent manner. This potentially occurs via the reduction in the probability of neurotransmitter release and may serve as a homeostatic measure to limit excessive excitation associated with the continuous sensory stimulation provided by enrichment.

**Figure 9:**
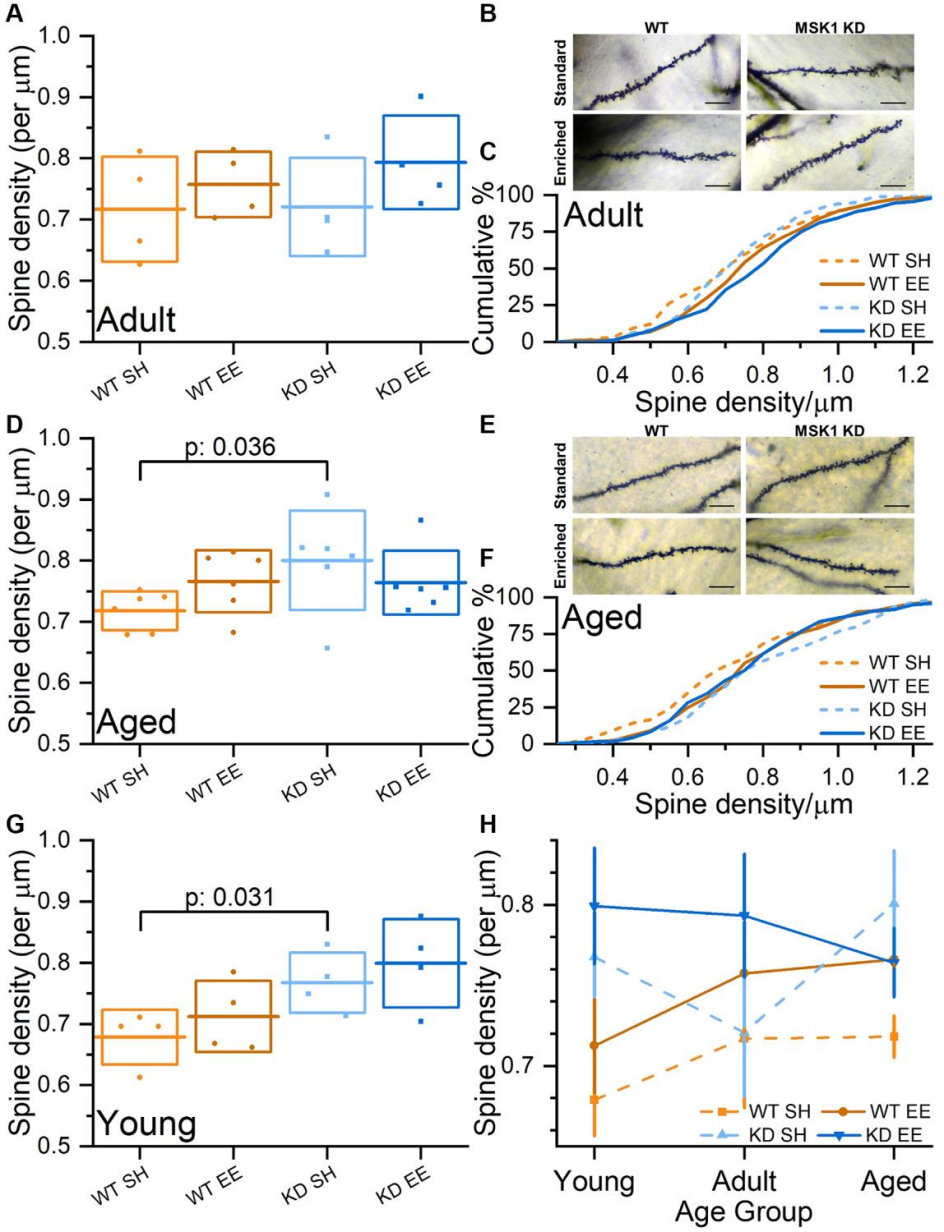
**Experience-, age- and MSK1-dependent effects on CA1 stratum radiatum dendritic spine density.** (**A**) In the Adult groups, when average CA1 stratum radiatum spine densities per animal are compared, there was a trend for enriched animals having greater spine density than standard-housed mice. (**B**) Representative Golgi images of secondary dendrites in stratum radiatum from CA1 pyramidal neurons across the four groups. Scale bars measure 10 μm. (**C**) Cumulative distribution of all spine density measurements across all groups showing rightward shifts of spine density in enriched groups. (**D**) In the Aged groups, when average CA1 stratum radiatum spine densities per animal are compared, there was a tendency for WT mice, but not MSK1 KD mice to show increased spine density after enrichment. (**E**) Representative Golgi images of secondary dendrites in stratum radiatum from CA1 pyramidal neurons across the four groups. Scale bars measure 10 μm. (**F**) Cumulative distribution of all CA1 stratum radiatum spine density measurements across all groups showing rightward shifts of spine density in enriched WT and standard-housed MSK1 KD mice. (**G**) Data from the Young groups are from Privitera et al., (2020) [36]. An across age group Univariate analysis with Genotype (WT or MSK1 KD), Treatment (SH or EE) and Age (Young, Adult and Aged) as independent variables revealed a main effect of Genotype (F(1,44) = 8.18, *p*: 0.006). A simple main Effects Analysis (Sidak correction) revealed a significant difference between standard-housed WT and MSK1 KD in the Young and Aged groups (F(1,44) = 4.96, *p*: 0.031 (**G**) and F(1,44) = 4.68, *p*: 0.036 (**D**), respectively confirming a previous report [34]. (**H**) A summary of CA1 stratum radiatum spine density changes across age, genotype and housing condition on a per animal basis (mean data from **A**, **D**, **G**). Enrichment consistently enhances spine density in WT mice. MSK1 KD mice typically have greater spine density, but the pattern over time, and the effects of enrichment are not correlated. In **A**, **D**, **G**, individual data points represent mean spine density values from individual animals (*n* = 4 (**A**, **G**) or 6 (**D**) mice), the horizontal line is the mean, and the box reflects ± 1 SD of the mean. In H data are presented as mean ± SEM. Additional CA1 stratum radiatum spine density data (per group and per animal) can be found in Supplementary Figure 3.

To confirm that the deficit in synaptic transmission, which has also been observed in the medial perforant path input to granule cells in the dentate gyrus in MSK1 knockout mice [59], was not due to a reduction in the density of dendritic spines, upon which excitatory synapses on CA1 neurons are located, we performed an age-, housing condition- and genotype-blind analysis of dendritic spines in stratum radiatum of Golgi-stained CA1 pyramidal neurons (Figure 9). Comparing the mean spine density in individual animals indicated that spine density is similar to (Figure 9A–9C; Adult mice), or indeed greater in MSK1 KD mice compared to WT mice (Figure 9D–9F; Aged mice), replicating observations made previously in younger mice (Figure 9G) [34, 36]. A comparison of CA1 stratum radiatum spine density across the lifespan (Figure 9H) demonstrated that, for the most part, standard-housed MSK1 mutant mice had greater spine density than their WT counterparts, and that enrichment enhanced spine density in both groups, most consistently in WT mice. This suggests that MSK1 is not required for the spine density increase provoked by enrichment. The data also suggest that MSK1 is either required for the pruning of dendritic spines, or that the elaboration of dendritic spines is a compensatory mechanism for reduced synaptic strength at individual spines.

**Figure 10:**
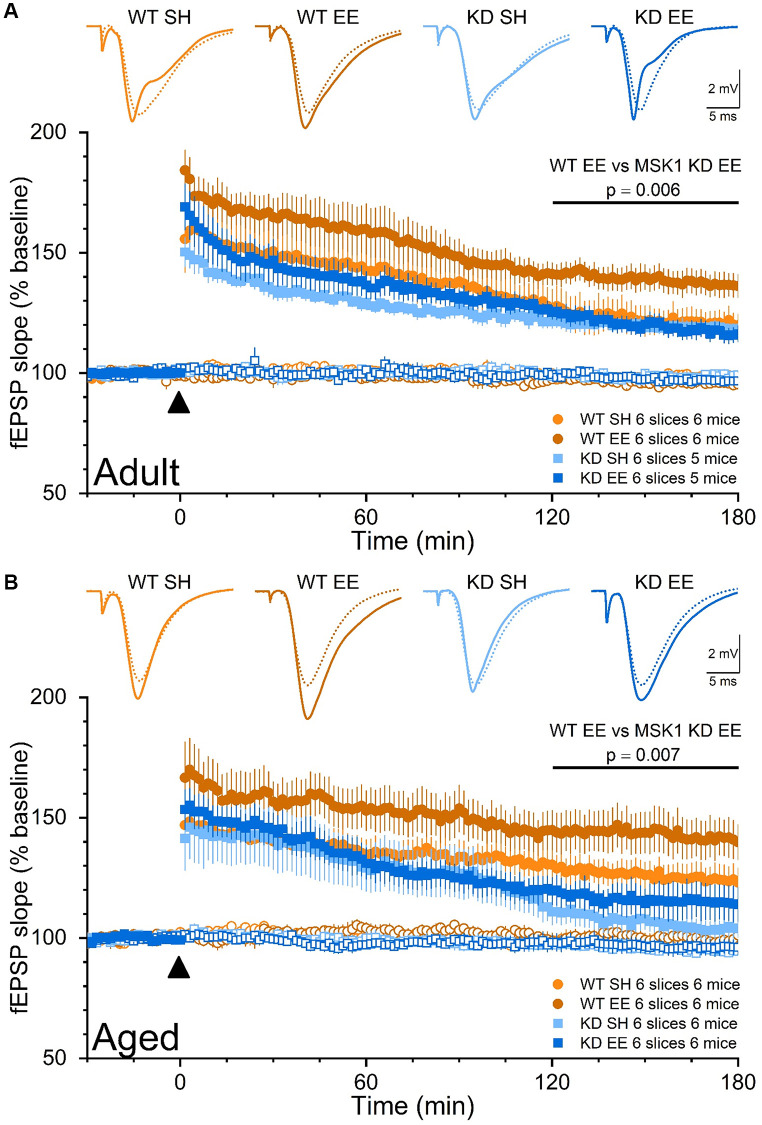
**The enrichment-induced facilitation of LTP in stratum radiatum of area CA1 is dependent upon the kinase activity of MSK1.** Dual pathway fEPSP recordings from stratum radiatum in area CA1 of WT and MSK1 KD mouse hippocampal slices. Filled symbols represent the pathway to which theta-burst stimulation (TBS) was delivered at time zero and denoted by the filled black triangle. Open symbols reflect synaptic transmission in the control, non-TBS, pathway, which served as a control for the viability of the slice over the recording period. Inset fEPSPs show baseline fEPSPs (broken lines) and fEPSPs 150 min after TBS (solid lines) from representative experiments. fEPSPs were stimulus-matched for amplitude prior to TBS delivery, and normalised with respect to average fEPSP slope measurements over the 30 min prior to TBS. (**A**) In the Adult groups the LTP analysis was calculated for the area under the curve (AUC) in the late phase (last 60 minutes) and showed a main effect of Genotype (F(1,20) = 6.20, *p* = 0.022). While there was no Genotype x Housing interaction (F(1,20) = 3.44, *p* = 0.078), a direct comparison between WTEE and KDEE showed a pronounced significant difference F(1,20) = 9.44, *p* = 0.006 while the same comparison between standard-housed mice was not significant (F(1,20) = 0.22, *p* = 0.660). In addition, while the difference between WT SH vs. WT EE was significant (F(1,20) = 7.05, *p* = 0.015), the same comparison between the MSK1 mutant mice did not reach significance (F(1,20) = 0.001, *p* = 0.970). (**B**) Similarly in the Aged groups the LTP analysis was calculated for the AUC in the late phase (last 60 minutes) and showed an even stronger effect of Genotype (F(1,20) = 13.51, *p* = 0.001) where WT mice had greater LTP, and statistical significance for Housing (F(1,20) = 4.34, *p* = 0.050). A direct comparison between WTEE and KDEE showed again a pronounced significant difference (F(1,20) = 9.22, *p* = 0.007). The lack of late LTP in the aged standard-housed MSK1 KD mice contrasted with the appreciable LTP seen in similarly housed WT mice, and was not improved by enrichment (F(1,20) = 1.07, *p* = 0.312). Data are presented as mean ± SEM.

To further understand the behavioural phenotype of MSK1 mutants we also investigated their ability to strengthen synaptic connections with an *ex vivo* double pathway analysis of LTP in stratum radiatum of area CA1 [36, 58]. In such experiments, one pathway served as an internal control for the stability of synaptic transmission, and the other pathway received stimulation to induce LTP. As a further criterion for the validity of the recordings, stimulus strength was adjusted to evoke fEPSPs of comparable amplitudes (~3 mV) across the 8 experimental groups in order that equivalent postsynaptic depolarisation was elicited. In Adult mice (Figure 10A) these experiments confirmed that CA1 LTP is no different between standard-housed WT and MSK1 KD mice, as observed previously in younger mice [36, 58], and in the dentate gyrus of MSK1 KO mice [59]. They also showed that LTP is selectively enhanced in enriched WT mice, with no effect on LTP in slices from MSK1 KD mice.

In Aged mice (Figure 10B), LTP in standard-housed mice was different between mutants and WT mice, with LTP returning to baseline within 3 hrs in MSK1 KD mice, but not in WT mice, where robust LTP was observed. A facilitation of LTP was seen after enrichment in MSK1 KD mice but failed to reach statistical significance above that of the standard-housed MSK1 KD mice. In contrast, the duration and extent of the enrichment-induced facilitation of LTP was much more pronounced in WT mice. These observations of enhanced LTP, especially in Aged enriched WT mice, may contribute to the enhanced performance in spatial memory tasks seen in these mice, while the LTP deficit and lack of appreciable facilitation of LTP in the MSK1 KD mice are likely factors in their poor performance on these tasks.

### MSK1 and experience regulate hippocampal gene expression

**Figure 11:**
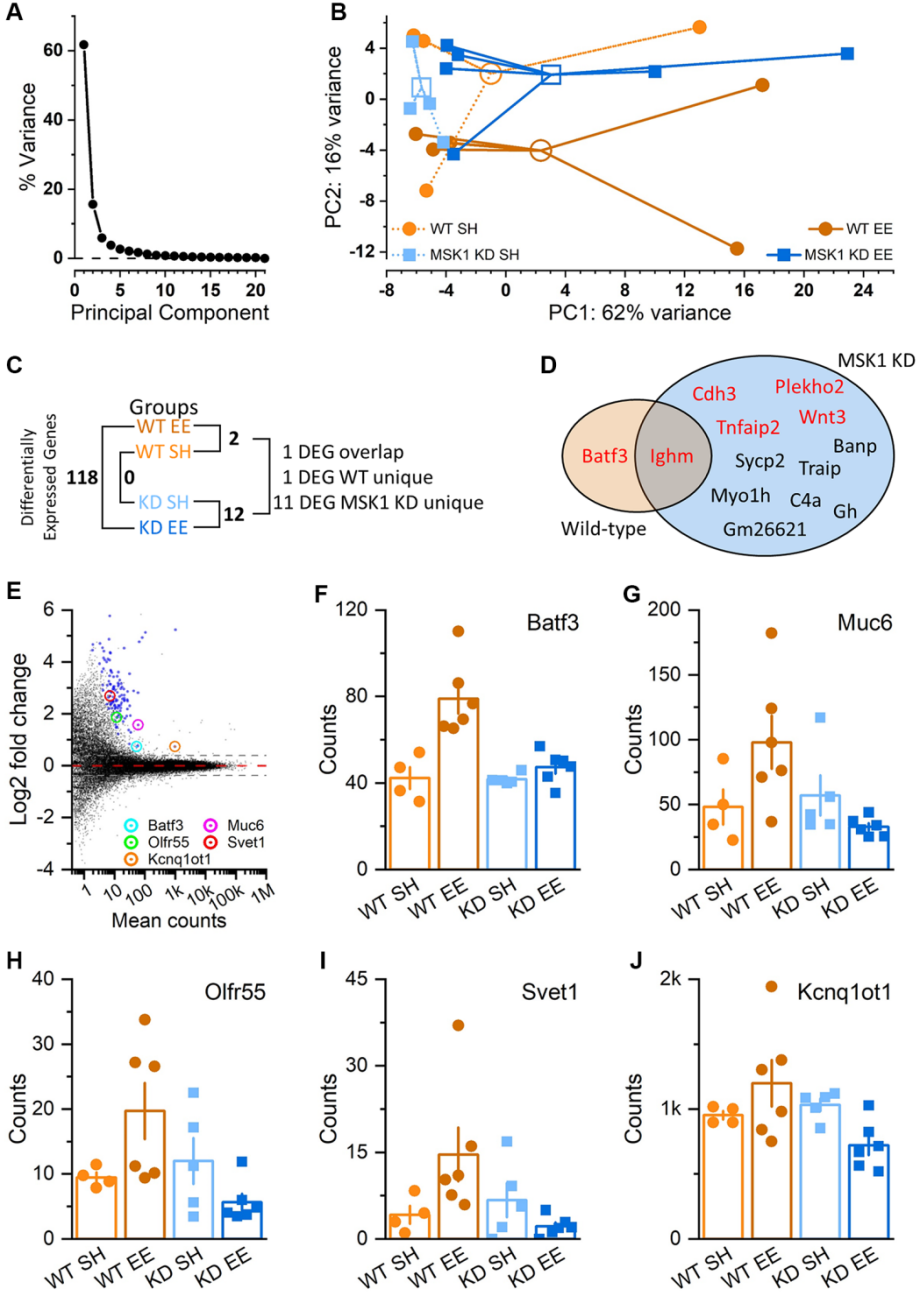
**Regulation of hippocampal gene expression by experience and MSK1.** (**A**) Scree plot of Principal Component (PC) variation as a percentage for the top 500 variance gene transcripts. One PC accounts for the majority of the variance (PC1; 62%), while the second PC (PC2) accounts for 12% of the variance. (**B**) PC analysis across groups and individual animals (filled symbols; 4–6 mice per group). Groups are identified by colour and are clustered around the arithmetic mean centroid (open symbol). The enriched WT group (WT EE) can be distinguished by migration along PC2. (**C**) Differential gene expression across groups, with the number of differentially-expressed genes (DEGs) given (Supplementary Data 1–7). (**D**) Venn diagram of unique and overlapping genes between WT and MSK1 KD mice. In red are genes that were upregulated by enrichment; in black text are those downregulated by enrichment in MSK1 KD mice (Supplementary Data 2 and 5). (**E**) MA plot of gene expression in enriched WT mice compared to that in enriched MSK1 KD mice. On the y-axis are plotted DESeq2 β-prior transformed log2 fold changes, with mean number of counts per group plotted along the x axis. Broken lines at ± 0.38 equate to a ± 1.3-fold change, with zero change indicated by the red broken line. Blue symbols show the 118 DEGs, while circled are five named genes with assigned functions (Supplementary Data 7). (**F**–**J**) Bar charts for each of the five genes showing individual counts for each animal in each group.

MSK1 regulates gene expression, including through the phosphorylation of CREB and histone H3 [40]. Since we previously observed an experience- and MSK1-dependent homeostatic downregulation of key MAPK signalling and plasticity-related genes and proteins in response to enrichment [36], we conducted an RNA-Seq analysis of gene expression in the hippocampi of Aged standard-housed and enriched WT and MSK1 KD mutant mice, since Aged mice benefitted most from the enrichment protocol. A principal component (PC) analysis (Figure 11A, 11B) revealed that the majority of the variance in gene expression (78 %) was captured by two PCs – PC1 (62 %) and PC2 (12%; Figure 11A). While there was overlap across the four groups particularly in PC1, PC2 separated WT enriched mice from the other three groups (Figure 11B). An analysis of differential gene expression (differentially-expressed gene; DEG) between the groups (Supplementary Data 1–7) showed that there were no DEGs between WT and MSK1 KD standard-housed animals, two DEGs between the two WT groups, and twelve DEGs between the two MSK1 KD groups (Figure 11C). Of these DEGs, one (Batf3) was unique and up-regulated in enriched WT animals; one (Ighm) was common to both and upregulated in enriched mice of both genotypes, and eleven were unique to the MSK1 KD mutant (Figure 11D). Of these eleven, seven were downregulated by enrichment and four were upregulated (Figure 11D).

Between the enriched mice, 118 DEGs were observed (Figure 11C), with all of them upregulated in the enriched WT mice (Figure 11E). The majority of these genes (Supplementary Data 7) were genes of unknown function, precluding their Gene Ontology (GO) classification. However, some (Batf3, Muc6, Olfr55, Svet1 and Kcnq1ot1) have had functions ascribed; these genes are indicated on the MA plot (Figure 11E) and the gene expression counts for each across the four groups of animals are provided (Figure 11F–11J, respectively). The role of these genes varies from a transcription factor regulating immune dendritic cell development (Batf3) [60], a long noncoding RNA involved in DNA methylation and transposon repression (Kcnq1ot1) [61], and developmental neuronal migration (Svet1) [62].

## DISCUSSION

Cognitive abilities decline across the lifespan and can be accelerated by neurodegenerative disorders such as Alzheimer’s disease. This can lead to dementia and increasing dependence on health-care systems, with the consequent impact on the individual, families and society [1]. Considerable interest exists in identifying strategies to alleviate the impact of ageing on human brain function, and a number of relevant factors revolve around lifestyle choices that may protect against the greater vulnerability of the human brain as ageing progresses [63]. Notably, several of these involve what might be regarded as environmental enrichment, in terms of being exposed to larger social groups, complexity/variety of experience, and physical activity [9, 64].

These features, which we have brought together, are provided in experimental environmental enrichment protocols that mimic holistic and translational approaches applicable to the ageing human. Such protocols have repeatedly been shown to have benefits on brain health and cognition across many models of both mental health and neurological disorders [14, 16, 54]. For example, pioneering studies in a mouse model of Huntington’s disease (R6/1 mice), a condition that had been described as 100 % genetic [65] showed that enrichment from 4 weeks of age spared brain tissue and dramatically delayed the onset and reduced the incidence of the motor signs associated with the mutation [26]. Potential mechanisms may range from an enrichment-induced stabilisation of the striatal phosphoproteome [66] to an influence on the gut microbiome [67]. In models of Alzheimer’s disease, early studies of enrichment showed a decrease in Aβ deposition in the brains of enriched mice, possibly due to an upregulation of the Aβ protease, neprilysin [28], and improved performance on Morris and radial water mazes, despite a potentially sex-related increase in amyloid load [29]. Mechanisms by which enrichment may reduce the impact of Alzheimer’s disease pathology may include stimulation of adult hippocampal neurogenesis or the enhancement of synaptic plasticity, through a range of intracellular signalling cascades and genes upregulated by enrichment [31]. These beneficial effects of an enriched environment have led to calls for the development of pharmacotherapeutics (“enviromimetics”) evoking the benefits of this enriched experience in those with congenital, acquired or age-related cognitive impairment [14, 32, 33].

Such endeavours require insight into the biochemical processes evoked by enrichment in order that compounds may be developed that target those particular pathways. Of note, BDNF-dependent signalling has repeatedly been implicated as mediating the beneficial effects of environmental enrichment [16, 31, 54], and one agonist for the BDNF TrkB receptor, 7,8-dihydroxyflavone, is reported to be efficacious in a range of experimental models of cognitive impairment [68–70]. Downstream of the BDNF TrkB receptor is MSK1 [16, 40], which we have shown previously in young animals is required for homeostatic synaptic plasticity [34], and both the expansion of the dynamic range of synapses and the full cognitive benefits that enrichment brings [36].

We now extend these observations across the lifespan with the view to establishing whether the BDNF/MAPK/MSK1 signalling cascade retains its relevance as a key transduction pathway for the benefits of environmental enrichment as age increases, particularly since the cellular and molecular mechanisms underlying the benefits of enrichment in late life have not been elucidated. We show that MSK1 retains its importance in converting positive experience into tangible synaptic and cognitive benefits well into old age, reinforcing the aged brain’s capacity to benefit from positive experience, MSK1’s prominence as a key player in the response to enrichment, and its potential as a target for enviromimetics.

### Experience induces age- and MSK1-dependent effects on exploratory and anxiety-like behaviours

In the present study we exposed separate groups of male C57Bl/6J mice to 10 weeks of enrichment prior to behavioural testing at two ages, at 6 months of age (the Adult group) and at 18 months of age (the Aged group). While mouse to human age comparisons are not a linear function [71], this timeline broadly equates to 4–6 human years of enrichment, starting from the late 20s and early 70s of human life, respectively [71], and approximates to the time spent by 70 year olds in care homes (between 4 and 6 years) [72]. Standard housing or enrichment continued for the remainder of the studies until the mice were 11.5 and 23 months of age, broadly equivalent to early 40s and late 70s in humans, respectively.

A recent survey of the enrichment literature showed that, in addition to a wide range of different environmental enrichment protocols, the ages at which animals are exposed to enrichment, and the duration for which enrichment occurs also varies widely across studies, with enrichment lasting anywhere between 2 and 20 weeks, with animals being tested between 6 and 100 weeks of age [16]. Thus, our protocol is not dissimilar from those reported previously, and had clear effects on behaviour, gene expression and spine density and synaptic plasticity. This may reflect the fact that enrichment started in the Adult and Aged groups at a time, before the median lifespan for the species and strain (32–33 months in male C57BL/6J mice [73, 74]), when the effects of ageing may be manipulable [75].

Ten weeks of environmental enrichment in Adult mice had tangible effects on behaviour. This was evident in the open field arena where enriched mice of both genotypes (WT and MSK1 KD) demonstrated less exploratory behaviour compared to their standard-housed counterparts. This was true for both the open field and open field + object components of the test, as we had previously observed in young (~4 months) mice raised form birth in enrichment [36], and by others in adult and aged rodents [76–79], and may be due to faster habituation to novelty by enriched animals [80, 81]. However, a genotype-dependent effect was observed upon exposure to the object, with reduced locomotor activity in the Adult MSK1 mutant mice (which was not seen previously in young mice). It is possible that the salience or potential threat posed by this object was not appreciated by the MSK1 KD mice, rendering them less inclined to increase motor activity in response.

As expected, Aged mice travelled less in the open arena, and the difference between enriched and standard-housed mice was only apparent within the first 10 mins of the trial. The lack of a clear differential thereafter (as per the Adult and young mice) could reflect a floor effect in generally lower levels of locomotion and physical activity in Aged animals [23]. Introduction of the object initially provoked increased locomotion in all groups except the standard-housed WT mice. The reasons for this are unclear, but could reflect a blunted response to salient stimuli in standard-housed Aged WT mice vs. increased anxiety-like behaviour in standard-housed Aged MSK1 mutant mice, and increased curiosity of the Aged enriched mice. This may be the case since the heatmaps suggested approaches to the object in the enriched mice. Thereafter in the open field + object phase only the enriched WT mice showed reduced locomotor activity, a pattern seen in young [36] and Adult enriched WT mice. This suggests that in Aged mice introduction of the object was capable of discriminating between the enriched genotypes, and may reflect a particular anxiety-like phenotype in the MSK1 mutant mice not completely ameliorated by enrichment.

Directly examining anxiety-like behaviour in the elevated plus maze showed that enrichment had no benefit in Adult mice, with enriched mice of both genotypes travelling as much into the open arms as the standard-housed mice. This is in contrast to the benefits on enrichment on reducing anxiety-like behaviour across genotypes in both young [36] and Aged WT and MSK1 mutant mice, all of which displayed increased exploration of the open arms of the maze, and which has also been observed in enriched aged rats [82]. This lack of effect of enrichment in Adult mice anxiety-like behaviour is somewhat surprising, but published reports are equivocal, with benefits in adult rats in some studies [83] but not in others [23], with equally equivocal observations in mice, where the duration of enrichment may be important [84, 85]. One potential explanation for the lack of effect of enrichment in Adult male mice may be a stronger instinct for self-preservation and the avoidance of potentially dangerous situations at a time when they are most likely to be sexually active. Thus, the drive to reproduce may lead to the avoidance of risk-taking when there is nothing obvious to be gained. In contrast, enriched Aged mice, well into their reproductive senescence period [71], did show reduced anxiety-like behaviour. From a translational perspective, enrichment strategies reducing anxiety or increasing confidence among the elderly may reduce loneliness and social isolation [86] or the fear of falling [87], and could lead to the mental and physical health benefits associated with engagement with social groups and physical exercise [64].

### The benefits of enrichment on hippocampus-dependent cognition become increasingly dependent upon MSK1 as ageing progresses

Spontaneous alternation is a test of hippocampus-dependent spatial working memory [88], and has been shown to be improved by enrichment, particularly as animals age [19, 23, 82, 89]. Similar to the observations made in young mice [36], enrichment had an overall beneficial effect on this form of cognition in Adult mice. However, in both young [36] and Adult mice, the benefits of enrichment were only statistically significant when the performance of WT mice was compared, suggesting that the kinase activity of MSK1 is necessary to fully transduce the enrichment experience into enhanced spatial working memory. In Aged mice, a clear interaction between genotype and housing was found, with only Aged WT mice showing benefits of enrichment, and the MSK1 mutant mice raised in standard and enriched housing behaving identically. This suggests that MSK1 is recruited during ageing to enhance spatial awareness in response to enrichment.

In the more cognitively-demanding Morris water maze for spatial reference memory, in which performance is also improved by enrichment [76, 82, 90–92], further distinctions in performance were made across age, housing and genotype. In Adult mice, learning of the platform location occurred across all groups, but with the clearest evidence for accelerated learning in enriched WT mice compared to all other groups, and with no difference in learning between standard-housed and enriched MSK1 mutant mice. This contrasts with the situation in young mice, where learning the water maze by enriched MSK1 KD mice was comparable to that of enriched WT mice [36]. Imposing further cognitive demands through reversal learning or the probe trial revealed further deficits in Adult MSK1 KD mice, in keeping with observations made in young mice [36]. Thus, the recruitment of MSK1 by enrichment for the learning of a spatial reference memory occurs in the Adult phase, while the dependence on MSK1 for more difficult cognitive tasks (cognitive flexibility, the persistence of memory) can be observed at an earlier age.

This age-dependence upon MSK1 reached its apogee in water maze experiments in Aged mice where the only evidence of strong learning of the platform location was found in enriched WT mice, with only weak, if any, learning of platform location occurring in the other three groups. This disparate learning precluded a reversal learning trial, but the probe trial showed that of all four groups, only the enriched WT mice performed above chance. Thus, the ability to learn the water maze declines with age, but can be ameliorated by exposure to an enriched environment [23, 82, 91]. Given spatial memory deficits that occur with human ageing [93], and the potential impact this has on, for example elderly pedestrians and vehicle drivers, enrichment strategies may improve the ability of the elderly to navigate environments, and enhance spatial awareness of both static and moving objects. Indeed, exposure to virtual environments have been shown to have benefits in improving spatial awareness among older people [94]. This suggests that plasticity remains within the ageing visuospatial system, and that it can be rehabilitated with exposure to appropriate stimulation, in a manner that is potentially dependent upon MSK1.

### Enrichment provokes age- and MSK1-dependent changes in the density of dendritic spines, synaptic transmission, and synaptic plasticity in stratum radiatum of area CA1

We have previously reported that synaptic transmission in area CA1 is impaired in young mice lacking the kinase function of MSK1 [36, 58], as it is in dentate gyrus of MSK1 knockout mice [59]. We replicated these observations in area CA1, and the absence of differences in axon excitability as measured by the fibre volley, in two further groups of mice of different ages, and reiterated the seeming reduction in the strength of synaptic transmission that enrichment provokes exclusively in WT mice [36]. The influence of enrichment on synaptic transmission has been studied by several groups, but the majority of studies report no significant differences in input/output synaptic transmission profiles between enriched and standard-housed animals [15, 16, 50]. One potential explanation is that this lack of change in the strength of synaptic transmission reflects a homeostatic adjustment to avoid potential overexcitation of neuronal networks, especially in the face of increased dendritic spine density, or that differences in enrichment protocols influence the outcome for basal synaptic transmission. For example, in a recent survey of electrophysiological studies after environmental enrichment [16], we found that the majority of studies reporting no change in basal transmission lacked one or more aspects of enrichment (social, complexity, exercise), whereas those studies showing increases of transmission included female animals. In the present study, all of the former, and none of the latter, were used.

Our studies, across three age groups, suggest that this homeostatic downregulation does occur to the point that electrically-evoked fEPSPs in enriched WT mice are subtly, but consistently, weaker than those in their standard-housed counterparts, and which may be due in part to a reduction in the probability of glutamate release, as evidenced by parallel increases in paired-pulse facilitation ratios. In contrast, no such downregulation of the fEPSP occurred in MSK1 KD mice, suggesting that MSK1 is necessary for this homeostatic adjustment in basal synaptic strength, as it is for homeostatic synaptic plasticity provoked by pharmacological activity deprivation or enhancement in cultured neurons [34].

These apparent decreases in electrically-evoked synaptic transmission provoked by enrichment in WT mice contrast with observations of enhancements in the amplitude of miniature excitatory postsynaptic currents (mEPSCs) observed after in enrichment in WT mice, but not MSK1 KD mice at both hippocampal CA1 [34] and neocortical synapses [37]. mEPSCs reflect the spontaneous and action potential-independent release of individual quanta of glutamate onto individual synaptic sites on postsynaptic neurones, and contrast with the electrically-evoked and action potential-dependent fEPSP, which reflects the sum of glutamate release across large numbers of synapses, and the amplitude of which will be influenced by spine density. Furthermore, different neurotransmitter vesicle pools are likely to contribute to synaptic transmission between neurons [95], and their recruitment may depend upon the nature of the stimulus. Thus, while quantal synaptic transmission may be enhanced by enrichment, at least in our studies (but see [96, 97]), this is not reflected at the population fEPSP level, as has been observed by others [15, 16, 50].

An analysis of the density of dendritic spines in area CA1, upon which these excitatory glutamatergic synapses form, showed that any deficits in synaptic transmission could not be accounted for by a decrease in spine density, either in enriched WT mice, or in the MSK1 KD mice, which showed an impairment in basal synaptic transmission under standard housing conditions, and reflected to some extent at the mEPSC level [34, 37]. On the contrary, spine density was consistently increased by enrichment in WT mice as we [34, 36] and others have observed previously (e.g., [16, 96]), and this was seen across the lifespan. However, MSK1 KD mice displayed both increased spine density under standard housing conditions and, depending upon the age of the animal, inconsistent responses to enrichment. This irregularity in spine density and the inconsistent influence of enrichment in the MSK1 KD mice suggests that the kinase activity of MSK1 is a necessary for either the formation or pruning of spines under basal conditions, and for the appropriate increase in spine density in response to enrichment. These observations potentially reveal another example of cellular homeostasis requiring MSK1, and is made all the more plausible given the activation of MSK1 by BDNF [39, 41, 58], and the importance of BDNF signalling in the growth and development of dendritic spines [98].

Whereas the majority of studies report no effect of enrichment on basal synaptic transmission, many studies reveal a facilitation of LTP by exposure to an enriched environment, particularly in hippocampal area CA1 [15, 16, 50]. This is especially the case if theta-burst stimulation (TBS) is delivered [16], a stimulation protocol that may avoid a ceiling effect on the level of potentiation, which may otherwise obscure an enrichment-induced facilitation. Alternatively, TBS may recruit the MAPK pathway [99], which is activated after enrichment [100, 101], but with some elements undergoing downregulation [102–104]; a downregulation that may occur in an MSK1-dependent and potentially homeostatic manner [16, 36].

In TBS LTP experiments where the stimulus strength was carefully adjusted to elicit basal fEPSPs of comparable amplitude across genotypes, we observed a facilitation of LTP in both Adult and Aged enriched WT mice, in keeping with observations made in a younger group of WT mice [36], and by others [15, 16, 21, 82, 105]. In contrast, no such facilitation was seen in Adult enriched MSK1 KD mice, where the level of potentiation in the 60 minutes from 2 to 3 hours after TBS was comparable to that observed in standard-housed WT and MSK1 KD mice. In Aged mice, differences in the MSK1-dependence of LTP did emerge, which were not seen in young [36] or Adult mice, in that LTP in Aged standard-housed mice decayed to baseline levels by 3 hours, a decline that was partially, but not significantly, attenuated by enrichment. These observations suggest that the dependence of LTP upon MSK1 increases as age advances, but that in parallel, or as a compensation, non MSK1-dependent processes can be recruited to partially preserve the synaptic response to enrichment. The MSK1-dependent enhancement of LTP by enrichment may occur via a lowering or restoring of the threshold for LTP induction that under normal circumstances rises with advancing age [106]. Thus, for a given stimulus, greater LTP is achieved, potentially via an experience- and MSK1-dependent tipping of the balance towards the NMDA-receptor dependent LTP seen in young animals that supports cognitive function [106]. Accordingly, it is plausible that the enrichment-enhanced LTP may contribute, at least in part, to some of the cognitive benefits associated with exposure to an enriched environment, not least in terms of spatial working and spatial reference memory, in the persistence of memory, and cognitive flexibility as reported previously for young mice [36].

### Hippocampal gene expression is regulated in an experience- and MSK1-dependent manner

The importance of gene expression for the acquisition of learning and the persistence of memory has long been appreciated [107–109]. Similarly, adaptation to novel experience invokes its own transcriptional response characterised by the induction of immediate early genes that coordinate subsequent transcriptomic changes [108]. Since MSK1 regulates gene expression, including via phosphorylation of the transcription factor CREB and chromatin remodelling through phosphorylation of histone H3 [40], its recruitment is likely to be important in the subsequent genomic response to an enriched environment. Indeed, we have previously shown that in young enriched WT mice, prolonged enrichment is associated with a homeostatic downregulation of key plasticity-related genes and proteins, some of which, e.g., for Arc and EGR1, occurs in an MSK1-dependent manner [16, 36]. We speculated that this may be necessary to either stabilise the neuronal networks underpinning enhanced cognition, or to provide a low background level of expression against which subsequent increases may have a greater impact. We thus conducted an RNA-Seq analysis on the hippocampal transcriptome of Aged WT and MSK1 mice raised under both standard and enriched housing conditions to establish if a transcriptome signature might emerge that may contribute to the enhanced cognitive properties of Aged WT mice.

In contrast to the down-regulation of gene expression that was prominent in young enriched WT mice compared to enriched MSK1 mutant mice, we found that there was an upregulation of gene expression between these two enriched groups in Aged animals. Given the delay (~10 weeks) between the introduction to the enriched environment and the sampling of hippocampal RNA, and at least two weeks after any behavioural procedures, any changes in gene expression are likely associated with maintenance of the enriched brain, as opposed to novelty associated with the new environment or cognitive testing. Most upregulated genes were of unknown function, precluding a gene ontology analysis, but several have been previously described and merit further consideration. Notably Batf3 was upregulated between standard-housed and enriched WT mice, and between enriched WT and MSK1 KD mice. Batf3 has been described as an important transcription factor regulator of dendritic immune cell expression, in particular of conventional type 1 dendritic cells (cDC1) [60]. These cells exert an important role in initiating innate and adaptive immune responses against infection [110] and brain tumours [111], and exert protective influences after cerebral ischemia [112]. It is possible that Batf3-upregulation reflects increased expression of these beneficial dendritic cells to mitigate some of the neuroinflammation associated with ageing [113], and which may manifest in some of the improvements in cognition and plasticity observed in enriched WT mice. Similarly, the upregulation of KCNQ1OT1 in Aged enriched WT mice compared to their MSK1 KD counterparts may reflect an anti-cellular ageing response. KCNQ1OT1 has recently been shown to enhance genome stability by transposon repression and in doing so reduce cellular senescence [61]. Since the loss of genome integrity through the failure of mechanisms to silence transposable elements may contribute to ageing-related cognitive decline [114], it is possible that the upregulation of KCNQ1OT1 may retard cellular ageing and hence protect the neuronal networks underpinning the enhanced cognitive repertoire of Aged enriched WT mice.

## CONCLUSIONS

We have shown that the kinase activity of MSK1 plays an important role in the regulation of basal synaptic transmission and the density of dendritic spines in area CA1 across the lifespan. The behavioural and synaptic plasticity consequences of this influence are not readily apparent under standard housing conditions where, for the most part mice lacking the kinase activity of MSK1 behave in a manner comparable to their WT counterparts [36, 58]. However, age and experience recruit MSK1 to regulate synaptic plasticity, spatial memory and gene induction to allow the full expression of an enriched experience to be manifest through enhanced cognition and LTP. This suggests that MSK1 may be both an important transducer of sensory experience into tangible genomic, morphological, cellular and behavioural manifestations of the experience, and a target for the development of enviromimetics for those suffering from the many forms of cognitive impairment that have been shown to benefit from enrichment.

## MATERIALS AND METHODS

### Animals

The MSK1 kinase dead (KD) mouse used in this study has been described previously, as have the breeding, housing and genotyping of the mutant mice and their wild type (WT) counterparts [34, 36, 58]. The generation of the MSK1 KD mouse (by Taconic Artemis) involved mutating Asp194 in the endogenous MSK1 gene to Ala (D194A). This results in the inactivation of the N-terminal kinase domain of MSK1, which is responsible for the phosphorylation of MSK1 substrates [115]. Confirmatory genotyping was conducted with PCR using the primers 5′-CGGCCATGTGGTGCTGACAGC-3′ and 5′-GGGTCAGAGGCCTGCACTAGG-3′, which gives 378 bp and 529 bp products for WT and targeted alleles, respectively. The mice used in this study were on a C57-Bl/6J genetic background after at least four backcrosses from the original C57-Bl/6n strain used to generate the mutant mice, and were kept as separate homozygous and WT lines derived from founder homozygous and WT breeders from an initial series of heterozygote crosses. To avoid genetic divergence of the two lines, subsequent backcrossing occurred when the founder mice had come to the end of their reproductive lifetime (typically three litters).

Mice were maintained under a 12/12 light/dark cycle, with lights on at 7.00 am, in a facility kept at 20–24°C. Mice were given ad libitum access to standard mouse chow and water. All animal procedures conformed with local, national and EU guidelines concerning the welfare of experimental animals. Behavioural studies were performed under the auspices of Home Office licence PPL 70/7821 granted to BGF. Male mice were used in this study to facilitate comparison with previous studies on MSK1 KD mice [34, 36, 58]. The mice have been deposited with the INFRAFRONTIER/EMMA repository at MRC Harwell, UK (https://www.infrafrontier.eu/emma/strain-search/straindetails/?q=13015).

### Environmental enrichment

For the provision of mice destined to remain in standard housing or be placed in an enriched environment, pregnant dams (E14-15, based on vaginal plugs) were placed individually in standard open top Tecniplast 1284L cages (365 × 207 × 140 mm; 530 cm^2^ floor area) to reduce sensory isolation during gestation. At weaning (P23-24), all females were removed, and the males (typically four) remained in an individually ventilated SH cage (Tecniplast 1285L; 396 × 215 × 172 mm; 542 cm^2^ floor area) until the enrichment protocol began. All male mice initially housed in SH cages were first randomly split into two cohorts destined to be used as Adult or Aged mice. Fifty percent of Adult mice SH cages were then randomly allocated to go into enrichment at 6 months of age while the Aged groups underwent the same treatment when they were 18 months old (see Figure 1 for details). Mice remained in their respective standard or enriched conditions for the remainder of their lives, up to 11.5 months for the Adult group, and 23 months for the Aged group, until all *in vivo* and *ex vivo* experiments were completed.

Environmental enrichment was provided as previously described [36] via the housing of WT and MSK1 KD mice in large individually ventilated rat cages (Tecniplast 1500U; 480 × 375 × 210 mm; 1500 cm^2^ floor area) containing bedding material, a cardboard tube, one running wheel and several plastic toys (tunnels, platforms, see-saws) and a metal ladder. To provide novelty, toys were moved around twice per week and new toys introduced once per week. Cage cleaning was done on Mondays for all standard and enriched cages. Toys in enriched cages were changed on Tuesdays and were moved around the enriched cages on Mondays and Thursdays. To keep disruption of the home environment to a minimum, sawdust and bedding were never changed at the same time as toys. To minimise disruptions to established hierarchies, during cage cleaning and behavioural testing all mice (standard and enriched) were removed to a different cage (standard cage size with one toy from the enriched cage for enriched mice) and then were returned together. This was effective in reducing within-cage aggression between mice. Standard housing (SH) comprised regular individually ventilated mouse cages (Tecniplast 1285L) with two to four mice per cage and containing bedding material and a cardboard tube.

Aged groups were composed of 8 males in enriched cages and 4 in standard cages. Similarly, Aged groups were composed of 8–10 males in enriched cages and 3, 4 in standard cages (Figure 1).

### Behavioural procedures

Mice used were scored for weight and against a battery of tests for neurological signs [53] before any behavioural experiment began. Different tests were conducted at weekly intervals to avoid one test influencing another (Figure 1).

#### Open field and novelty-induced locomotion

These tests were run as two consecutive stages of the same experiment. Four open field boxes (Ugo Basile; 44 × 44 × 44 cm) were placed inside the empty water maze arena to form a square. Four mice were tested simultaneously. Each mouse was singly released in each box and tracked. Exposure to the open field lasted for 1 hour after which, for the novelty stage of testing, an object (a 50 ml plastic vial; Falcon) was secured upside-down to the centre of the arena and the mouse was tracked for an additional hour.

#### Elevated plus maze

An 8-radial arm maze for mice (Ugo Basile) was placed within the empty water tank and raised 60 cm from the tank base. Four of the eight arms were kept open to form a plus shape; two of the arms had walls while the other two (opposite one another) were without walls. Each mouse was individually released in the centre of the maze and video tracked for 10 minutes.

#### Spontaneous alternation for spatial working memory

An 8-radial arm maze for mice (Ugo Basile) was placed within the 180 cm wide tank used for the water maze. Four out the eight arms (with walls) were kept open to form a cross while the other four arms were kept closed. Each mouse was individually released in the centre of the maze and video tracked for 10 minutes. The sequence of arm entries was scored. A correct alternation was considered when a mouse made one repetition over five entries [116].

#### Water maze reversal learning protocol for cognitive flexibility – Adult mice groups

An inter-trial interval of 120 s over 4 daily trials was employed. The pool was filled daily with fresh water, which was made opaque by the use of full fat UHT milk.

Stage 1 (day 1 and 2) Habituation: each mouse was placed on a 20 cm diameter platform located in the centre of a 180 cm diameter pool filled with opaque water (28°C) and was allowed to observe the environment for two minutes. The pool was surrounded by curtains which did not allow the distal visual cues to be seen. Water level was ~1 cm above the top of the platform. Each mouse then received 3 consecutive trials (each with a different starting point) where it was left free to swim in the pool for a maximum of 90 seconds and then placed on the platform and left there for 30 sec.
Stage 2 (day 3 and 4) Visual Cue: The platform was placed in the centre of the pool and a visible object was placed upon it (yellow TV toy 6 × 6 × 5 cm). Each mouse received 4 consecutive trials (different cardinal starting points) where it was left free to swim in the pool for a maximum of 90 s. Water level was ~1 cm above the platform surface. Water was kept at 26°C. The pool was surrounded by curtains which did not allow the distal visual cues to be seen.
Stage 3 (day 5 to 8) Training: Curtains were removed. Water was kept at 26°C. The platform was placed in the centre of the South-East or North-West quadrant and kept constant for any given mouse. Water level was ~1 cm above the platform surface. Each mouse received 4 trials (different starting points) where it was left free to swim in the pool for a maximum of 90 seconds and then left on the platform for 30 sec.
Stage 4 (day 9 and 10) Reversal Learning: The platform was placed in the quadrant opposite to that used during Training. All other parameters as per Stage 3.
Stage 5 (day 11) 24 hrs delay Probe trial: Water was kept at 26°C. The platform was removed, and distal spatial cues were present as per previous the stage. Each mouse received a single 90 sec trial. Starting point was distal to the location of the platform during Reversal learning; e.g., if platform was South-East starting point was North.

#### Water maze for spatial reference memory- Aged mice groups

Stage 1 Habituation (day 1 and 2): as described above.
Stage 2 (day 3 to 5) Visual Cue: as described above.
Stage 3 (day 6 to 9) Training: as described above.
Stage 4 (day 10) 24 hrs delay Probe trial: as described above in stage 5.

As outlined in Figure 1, mice used for these experiments were experimentally naïve with respect to the water maze, but had undergone open field without and with the inclusion of an object, the elevated plus maze and spontaneous alternation.

All behavioural tests were video-tracked and analysed using AnyMaze 4.99 video tracking system.

All the behavioural experiments were conducted blind to genotype.

#### Ex vivo morphological and electrophysiological analyses

At least two weeks after the end of behavioural testing (Figure 1) male WT and MSK1 KD mice were killed by cervical dislocation in accordance with the UK Animals (Scientific Procedures) Act 1986 and with local Animal Welfare and Ethical Review Board approval, and as previously described [36].

#### Analysis of dendritic spine density

Mice brains were processed with the FD Rapid Golgi Stain kit (FD NeuroTechnologies, Inc.) in accordance with the manufacturer’s protocol. Impregnated brains (4 or 6 per group) were sectioned with a vibratome (coronal sections; 200 μm thick) stained and mounted. Dendritic spines on the secondary branches of apical dendrites of hippocampal CA1 neurons were counted. Spine count was conducted blind to genotype and housing condition. ImageJ software was used to measure dendritic length and the numbers of spines on each dendritic segment. Images for spine density analysis were captured with a 40× objective on a Zeiss Imager 2 AXIO microscope.

#### Hippocampal slice preparation and extracellular recordings

After decapitation, the brain was rapidly removed from the skull and kept covered with ice cold aCSF during the dissection. Two hemispheres were obtained and glued on their temporal side, submerged in ice cold aCSF bubbled with 95% O_2_/5% CO_2_. Parasagittal hippocampal slices (400 μm) were cut and transferred to an interface recording chamber, where they were incubated for at least 2 hrs prior the recording and where they remained for the duration of the experiment. The temperature of the aCSF was set at 31° and the flow rate was ~1.5 ml/min. The aCSF used for the incubation and recording of slices contained in mM: 124.0 NaCl, 4.4 KCl, 1.0 Na_2_HPO_4_, 25.0 NaHCO_3_, 2.0 CaCl_2_, 2.0 MgCl_2_, 10.0 D-glucose. aCSF was bubbled with 95% O_2_/5% CO_2_; pH 7.4. During slice preparation the aCSF contained a higher concentration of Mg^2+^ (8 mM). All salts used in the aCSF were obtained from either Fisher Scientific or Sigma-Aldrich.

Recordings of field excitatory postsynaptic potentials (fEPSPs) were made using an aCSF-filled glass microelectrode placed in stratum radiatum of area CA1. Two concentric bipolar stimulating electrodes (CBBRC75, FHC) were placed either side of the recording electrode. This allowed alternating recordings to be made from two independent but convergent afferent Schaffer collateral/commissural fibre pathways. Each pathway was stimulated every 90 s with a monophasic pulse of 0.1 ms duration. Pathway-independence was assessed via a crossed paired-pulse facilitation protocol (at 50 ms interpulse interval). Independence was accepted when facilitation of the second pulse was ~10 % or less. To assess basal synaptic transmission, stimulus input/fEPSP slope output curves were constructed over the range of 20–300 μA. A minimum of four fEPSPs were averaged to yield a fEPSP slope measurement at each stimulus intensity. Paired-pulse facilitation (PPF), a commonly used index of the probability of neurotransmitter release, was assessed over an inter-stimulus interval of 50–350 ms, with the average of at least two fEPSPs yielding the slope measurement at each paired-pulse interval. In all experiments both pathways in each slice were tested for input-output and PPF profiles and all were taken into consideration in subsequent analyses.

For the LTP experiments a stable baseline of at least 30 mins was achieved on both pathways before theta-burst (TBS) was delivered to one pathway. TBS consisted of bursts of 4 stimuli at 100 Hz with 10 such bursts comprising a train. Each burst within a train was separated by 200 ms. Trains were repeated 3 times with an inter-train interval of 20 seconds. The second pathway was not subject to TBS and served as a control for the stability of the recordings. Experiments were excluded from analysis if the control pathway deteriorated by more than 10 % within the 3 hours post-TBS monitoring period.

Given the deficit in basal synaptic transmission observed in MSK1 KD mice, care was taken to match the baseline strength of synaptic transmission, which involved adjusting the stimulus intensity to yield fEPSPs of ~3 mV across all groups. Electrophysiological recording parameters and the analysis of fEPSPs were under the control of WinLTP program [117]. Experiments were interleaved and performed blind to the identity and housing condition of the mice, which was revealed only after the experiments had been analysed, with genotype confirmed with post-hoc genotyping as required.

### RNA-seq analysis

#### RNA-Seq

Hippocampal RNA was prepared from individual hippocampi from experimentally naïve mice of 21–23 months of age. Samples were prepared and analysed blind to the two genotypes and two housing conditions, 6 samples for each of the 4 experimental groups (Supplementary Table 1).

#### RNA extraction and library preparation

Hippocampi were extracted and then rapidly homogenized in Trizol (Invitrogen, #15596018). Precipitation of total RNA was conducted using isopropanol following the manufacturer’s protocol before DNaseI treatment. The quality of the extracted RNA was checked by Nanodrop and Qubit 4 fluorimeter (Invitrogen).

TruSeqv2 (Illumina) LS protocol was then used to prepare mRNA libraries, which was conducted in-house by the School of Life Sciences Genomics Facility. Briefly, pull-down of poly-A mRNA was performed using poly-T magnetic beads. The RNA was fragmented and random hexamers used to prime fragments prior to first-strand synthesis. Blunt end repair was performed with a 3’ to 5’ exonuclease following second-strand synthesis, and 3’ ends were adenylated. cDNA was then ligated to adaptors. Quality checking for the 24 library samples was performed on a Qubit 4 fluorimeter (Invitrogen) and a 2100 bioanalyser (Agilent) before being multiplexed 6 samples to a lane and 150bp paired-end sequencing performed on an Illumina HiSeq 4000. A mean of 62.11M reads per sample were obtained (Supplementary Data 8).

### Analysis pipeline

#### Quality control and trimming

Raw fastq files for each sample were initially quality checked using FastQC (v0.11.3) [118]. Adaptor contamination removal was performed with Skewer (v0.2.2) [119], using Illumina TruSeq v2 adapter lists, which included reverse complements and theoretical PCR product. Adapter contamination removal was confirmed using FastQC. Further trimming of the Fastq files was also conducted if mean base quality fell below 10 (4bp window). Only reads >50bp were kept for alignment. Paired fastq files for each sample (forward and reverse) were aligned to the mouse genome (GRCm38) using STAR aligner (v2.5) [120] and annotated (GRCm38.87). Mean read alignment was 91.83% for samples that met quality control criteria (21/24) and were included in the analysis (Supplementary Data 8).

IGV (v2.3.65) [121] was used to inspect read alignment and verify the presence of the point mutation (D194A) at the MSK1 gene locus [34] in samples belonging to MSK1 KD group (Supplementary Figure 4). SeqMonk [122], was then used to generate read probes for each sample and examine read alignment within ribosomal RNA, mitochondrial genes, and exons/introns (Supplementary Figure 5). Read alignment within individual genes was then quantified using HtSeq (v0.6.1p1) [123], utilising parameters specifying unique read alignment, that reads were un-stranded, and that only reads with a minimum average PHRED quality score of 10 were counted.

Ribosomal (rRNA) contamination was observed in samples 4 and 7 (~23% of reads aligned to rRNA in each sample) indicating poor polyA RNA purification for these samples (Supplementary Figure 5). Intra-group sample variation (calculated using Pearson’s correlation coefficient) was observed to be low (<0.9) for samples 4, 6 and 7, indicating excessive variance compared to other samples within the group (Supplementary Figures 6, 7). Samples 4, 6 and 7 were therefore excluded from further analysis, as reflected in the final sample table (Supplementary Table 2; Supplementary Data 8), which was used for subsequent differential gene expression testing. A summary of the number of differentially-expressed genes (DEGs) across the groups can be found in Supplementary Data 1, and gene lists for each differential gene expression comparison are included in Supplementary Data 2–7.

R (4.1.0) [124] was used to perform principal component analysis utilising the DEseq2 package (v1.32.0) [125]. A log2 fold-change cut-off of 0.38 and a Benjamini-Hochberg corrected *p* value threshold of 0.05 were used to determine significance for differential gene expression, conducted using the Wald test statistic. The topGO package (v2.44.0) [126] was used to perform gene ontology enrichment analysis against ontology org.Mm.eg.db (v3.13.0) [127]. The “classic” algorithm [126] and Fisher’s exact test (Benjamini-Hochberg corrected) were used for enrichment scoring with an adjusted *p* value of < 0.05 considered significant. Unless stated, default parameters were utilised for all tools. Scripts used for these analyses can be found on Github.

### Statistical analysis

Statistics were computed by IBM SPSS 27 using two-tailed one or two-way analysis of variance (ANOVA) with genotype and housing condition as the two between group factors and day of training, time-point or stimulus strength as within factor as appropriate, with simple main effects or main effects as the post-hoc comparison. In addition to significant housing x genotype interactions, planned comparisons regarding the individual effects of genotype or housing were conducted and reported, as per [36]. The level of significance was taken to be *p* < 0.05 (α = 0.05). Exact *p* values are reported where *p* ≥ 0.0001. For lower values, *p* < 0.0001 is reported; these very low *p* values ranged from *p* = 1.7 × 10^−5^ to *p* = 1.59 × 10^−270^. Data are reported as mean ± SEM and bar graphs display individual data points.

### Data availability

The RNA-Seq data has been posted to the Gene Expression Omnibus database: https://www.ncbi.nlm.nih.gov/geo; Accession code: GSE235037. Other data will be made available upon reasonable request.

## Supplementary Materials

Supplementary Figures

Supplementary Tables

Supplementary Data 1-8
