# Supplementary material for: MSK1 is required for the beneficial synaptic and cognitive effects of enriched experience across the lifespan
Source: Aging (Albany NY). 2023 Jul 10;15(13):6031–72. doi: 10.18632/aging.204833 (PMC10373962; doi:10.18632/aging.204833)
Supplement: Supplementary Figures [file aging-15-204833-s001.pdf]

SUPPLEMENTARY FIGURES

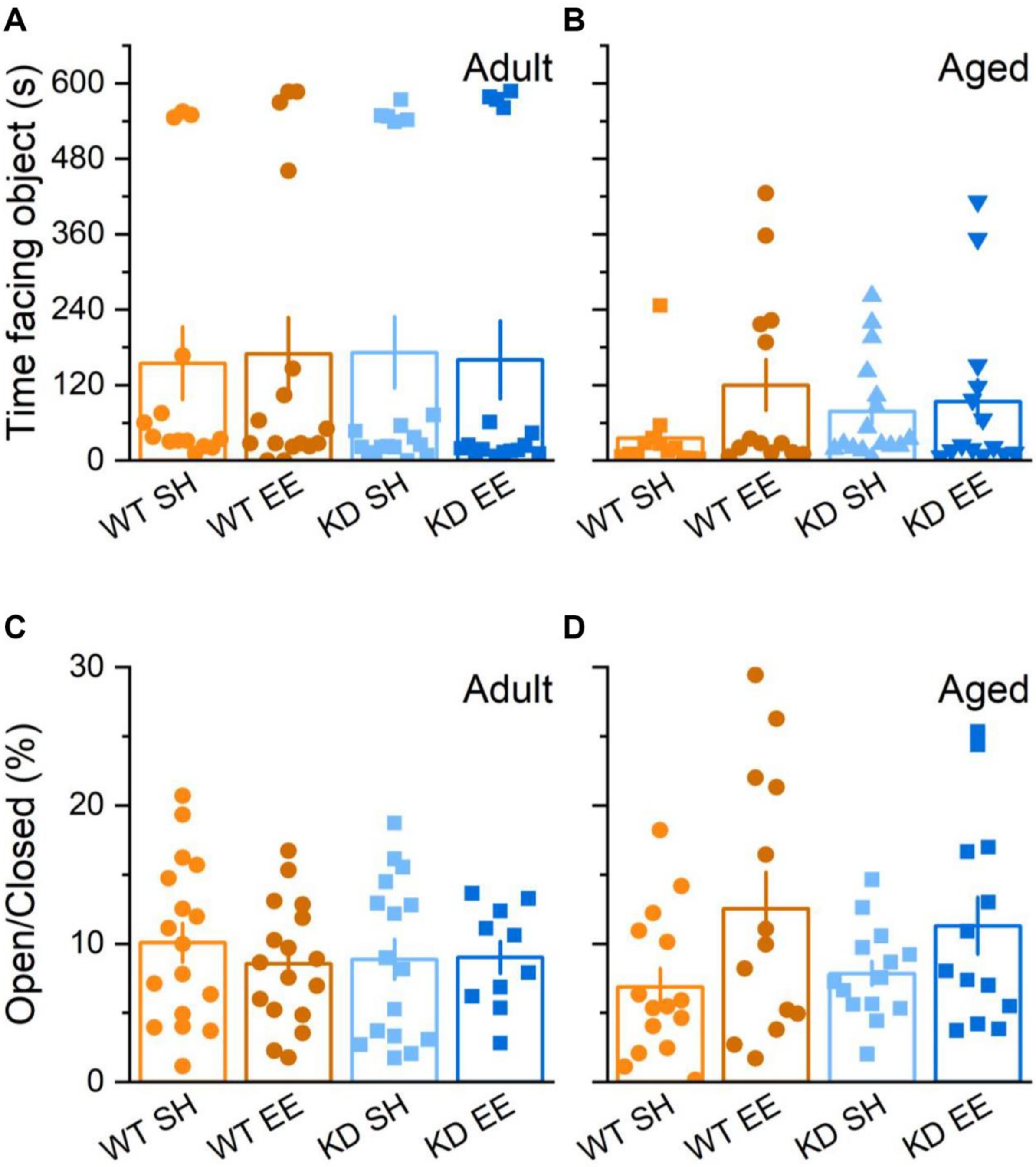

Supplementary Figure 1. Open field and elevated plus maze behaviour. (**A**, **B**) show the time spent by each Adult and Aged mouse facing the newly-introduced object (a 50 ml Falcon tube) in the Open Field arena, respectively, in the 10 minutes after its introduction. There were no significant differences or interactions in either the Adult mice (Genotype: $F(1,60) = 0.01$, $p = 0.952$; Housing: $F(1,60) = 0.01$, $p = 0.977$; Genotype x Housing: $F(1,60) = 0.05$, $p = 0.822$) or in the Aged mice (Genotype: $F(1,52) = 0.08$, $p = 0.780$; Treatment: $F(1,52) = 2.84$, p: 0.098; Genotype x Housing: $F(1,52) = 1.34$, $p = 0.252$). (**C**, **D**) show the % time spent in the open arm of the elevated plus maze in Adult and Aged mice, respectively. There were no significant difference or interactions in the Adult mice (Genotype: $F(1,56) = 0.07$, $p = 0.786$; Housing: $F(1,56) = 0.25$, $p = 0.620$; Genotype x Housing: $F(1,56) = 0.37$, $p = 0.544$). In the Aged mice, there was an effect of Housing ($F(1,51) = 6.35$, $p = 0.015$), but no effect of Genotype ($F(1,51) = 0.01$, $p = 0.943$) and no Genotype x Housing interaction ($F(1,51) = 0.37$, $p = 0.544$). The Simple Main Effects analysis revealed only a significant difference between WT SH and WT EE ($F(1,51) = 4.98$, $p = 0.030$). The scale and titles in A and C apply to B and D.

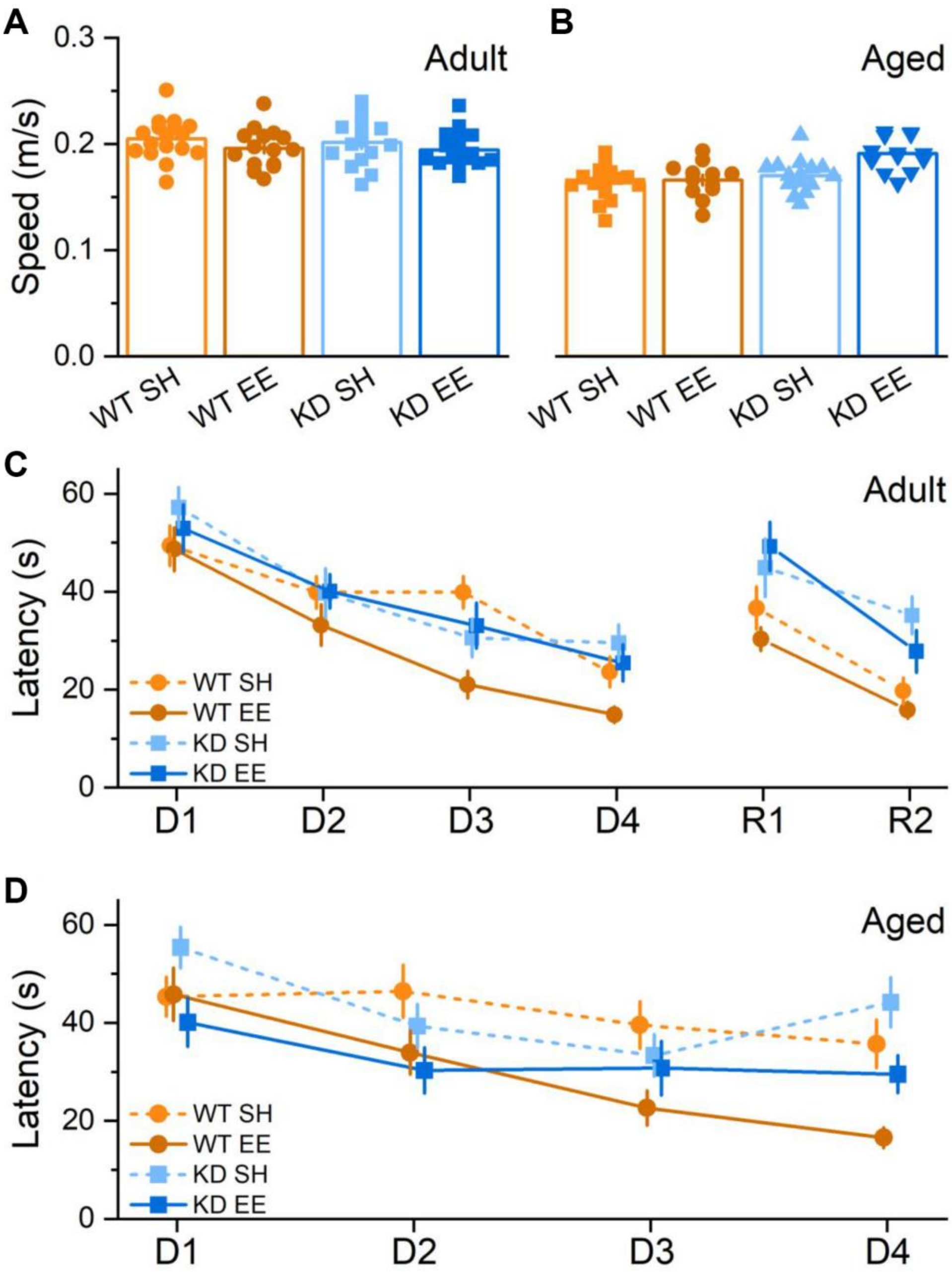

Supplementary Figure 2. Swimming speed and latency to platform in the Morris Water Maze. (**A**, **B**) average swimming speed of Adult and Aged mice, respectively. Swimming speed was significantly lower in Aged mice compared to Adult mice ($F(1,110) = 64.25$, $p < 0.0001$). In Adult mice (**A**), there was no significant effects of Genotype ($F(1,59) = 0.28$, $p = 0.600$) or Housing ($F(1,59) = 2.61$, $p = 0.111$), and nor was there a Genotype x Housing interaction ($F(1,59) = 0.04$, $p = 0.850$). In Aged mice (**B**) there was an effect of Genotype ($F(1,51) = 13.37$, $p = 0.0006$) and Housing ($F(1,51) = 7.15$, $p = 0.010$), and a Genotype x Housing interaction ($F(1,51) = 4.38$, $p = 0.041$). A Simple Main Effects analysis revealed that this was reflected in higher swim speeds in enriched MSK1 KD mice compared to both enriched WT mice ($F(1,51) = 15.14$, $p = 0.0003$) and standard-housed MSK1 KD mice, ($F(1,51) = 11.61$, $p = 0.001$). (**C**) Latency to platform during training and reversal learning in Adult mice. There was a Main effect of day of training (D1 – D4; $F(3,177) = 54.01$, $p < 0.0001$) and a Main effect of Genotype ($F(1,59) = 10.75$, $p = 0.002$). A Simple Main Effects analysis revealed a significant difference between WT and MSK1 KD on day 3 and day 4 ($F(1,59) = 5.83$, $p = 0.019$ and $F(1,59) = 5.53$, $p = 0.022$, respectively). During the Reversal stage (R1 and R2) there was a Main effect of day of training ($F(1,59) = 42.37$, $p < 0.0001$) and a Main effect of Genotype ($F(1,59) = 21.14$, $p < 0.0001$). A Simple Main Effects analysis revealed significant differences between WT and MSK1 KD in the EE group on R1 ($F(1,59) = 8.55$, $p = 0.005$) and on R2 ($F(1,59) = 6.38$, p = 0.014), and within the SH group on R2 ($F(1,59) = 10.18$, $p = 0.002$). (**D**) In the Aged group, There was a Main effect of Housing ($F(1,51) = 18.87$, $p < 0.0001$). A Simple Main Effects analysis revealed significant differences between SH and EE in the MSK1 KD group at D4 ($F(1,51) = 5.56$, $p = 0.021$) and in the WT group at D3 and D4 ($F(1,51) = 6.44$, $p = 0.014$, and $F(1,51) = 9.20$, $p = 0.004$, respectively). The higher swim speed of the Aged enriched MSK1 KD mice (**B**) may have influenced the time taken to reach the hidden platform and thus latency might not provide the most accurate reflection of cognitive ability.

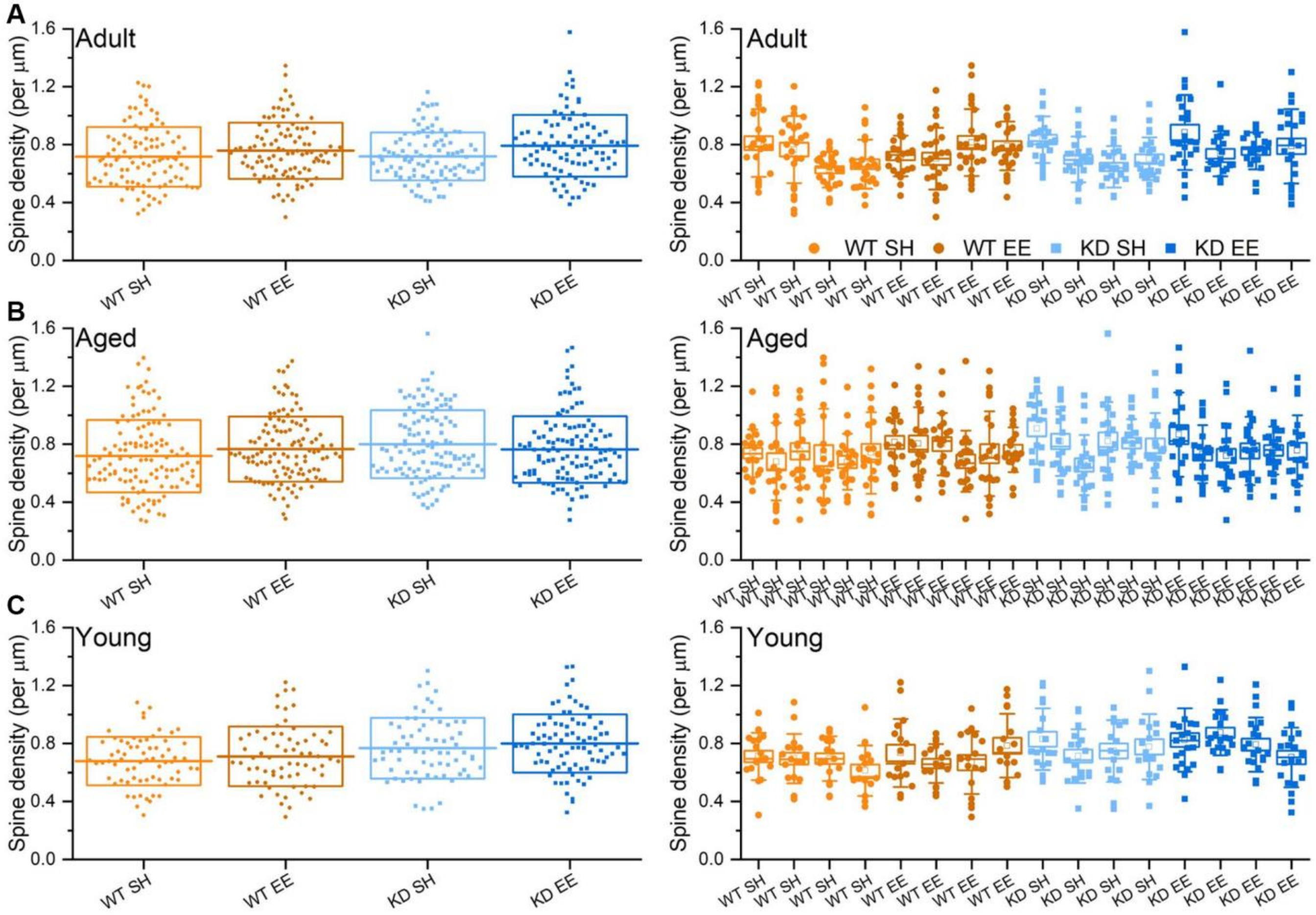

Supplementary Figure 3. Individual spine density measurements across groups and animals. Left hand panels depict all spine density measurements across animals grouped by genotype and housing for (**A**) Adult, (**B**) Aged and (**C**) Young animals (from Privitera et al., 2020). Right hand panels show corresponding spine density measurements on a per animal basis according to genotype and housing.

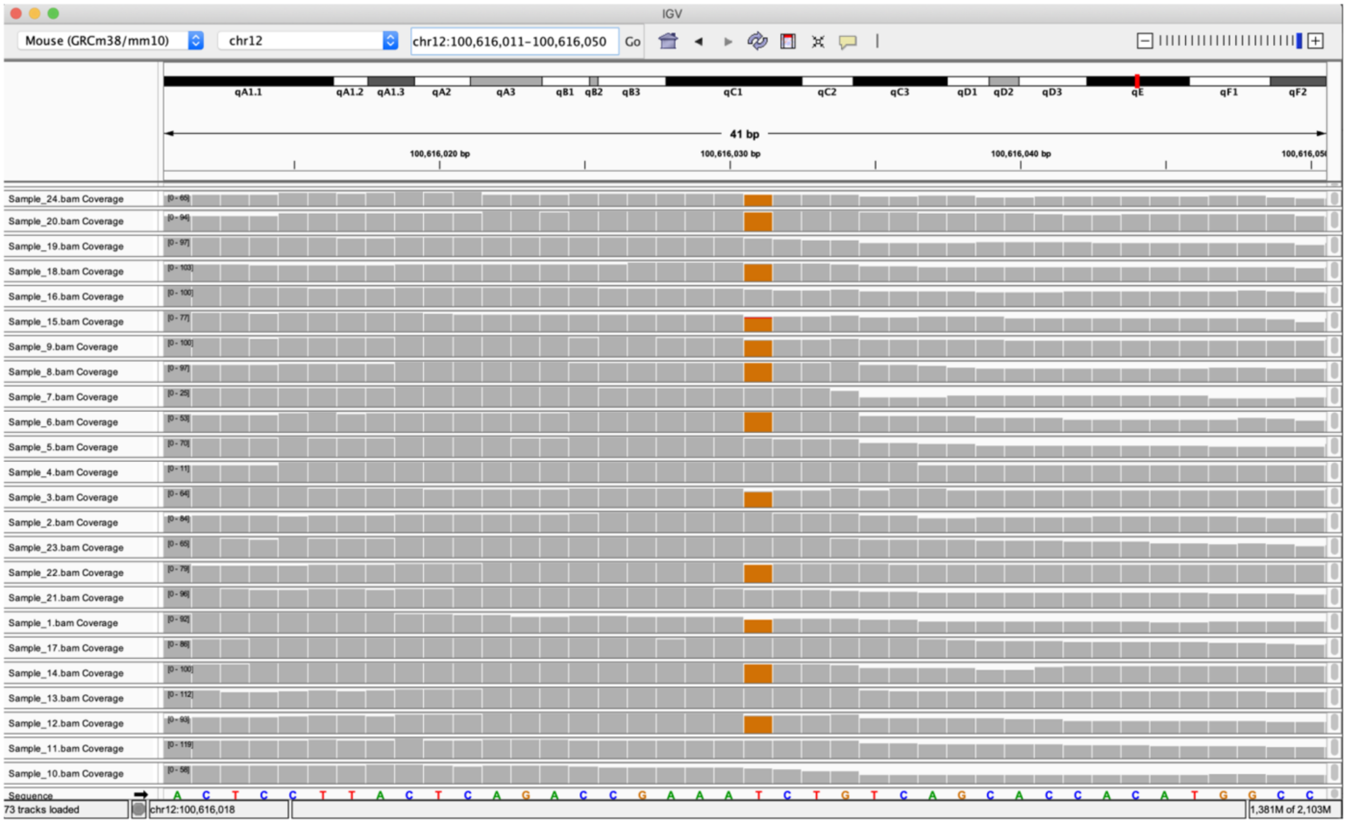

Supplementary Figure 4. Screenshot from IGV Browser displaying alignment of all samples to Grcm38.87 reference genome, at base pair resolution at the MSK1 gene locus on chromosome 12. At base 100,616,031, a mis-match between the aligned reads and the genome can be observed (orange bases), due to an D194A point-mutation (Thymine to Guanine) in the MSK1 kinase domain (as previously described in Correa et al., 2012). Grey columns indicate a matching base pair, orange a mismatch. Heights of each base for each sample are based on the amount of reads overlapping each base pair. Note the y axis for each sample is auto-scaled to individual sample expression.

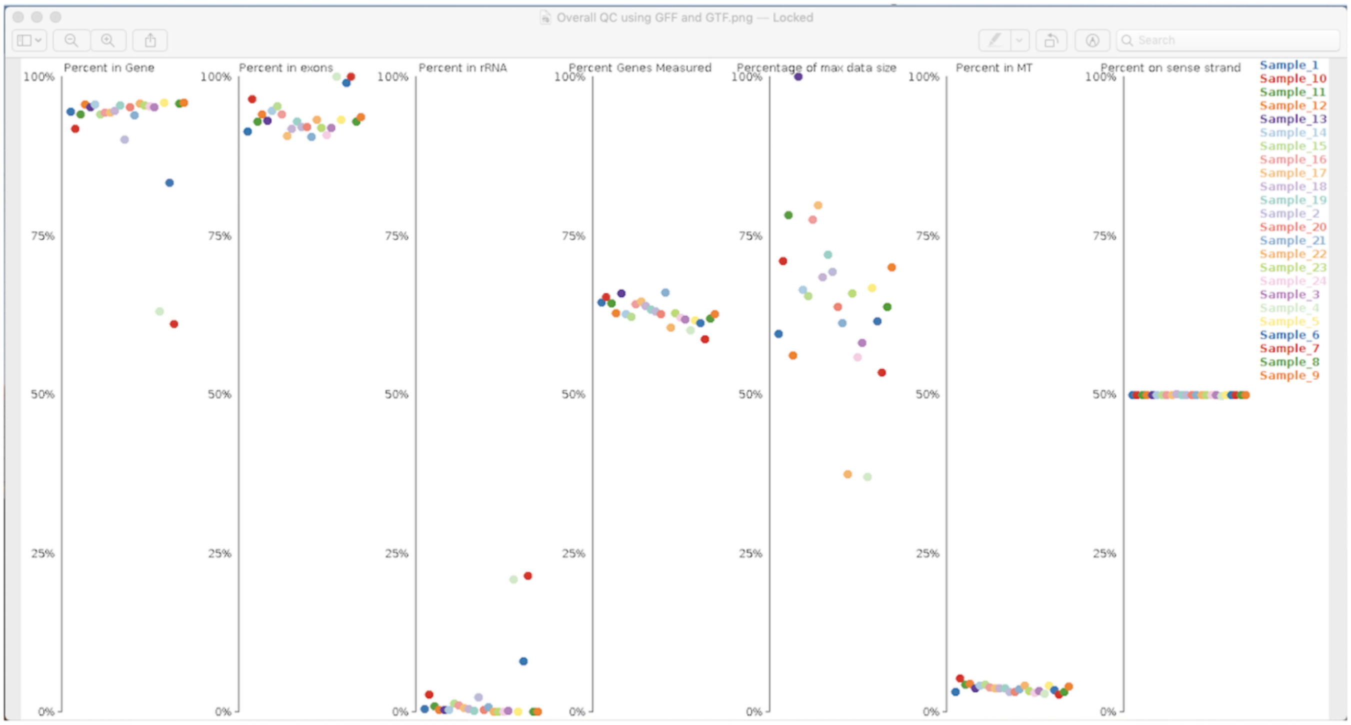

Supplementary Figure 5. RNAseq QC plot generated by SeqMonk. Reads are mapped to specific genome features as a percentage of total reads. From left to right: % reads mapping to gene features, % mapping to exons, % mapping to ribosomal RNA, % of genes with reads mapping to them, % when comparing reads in sample to reads of largest sample, % reads mapping to mitochondrial (non-genomic) RNA, % reads on sense strand. All samples are colour-coded, and the left-most sample in a column corresponds to the top-most sample in the legend. Note the large degree of mapping to rRNA in samples 4, 6 and 7; 20–25% for sample 4 and 7, ~10% for sample 6. This indicates poor quality RNA purification during sample library prep.

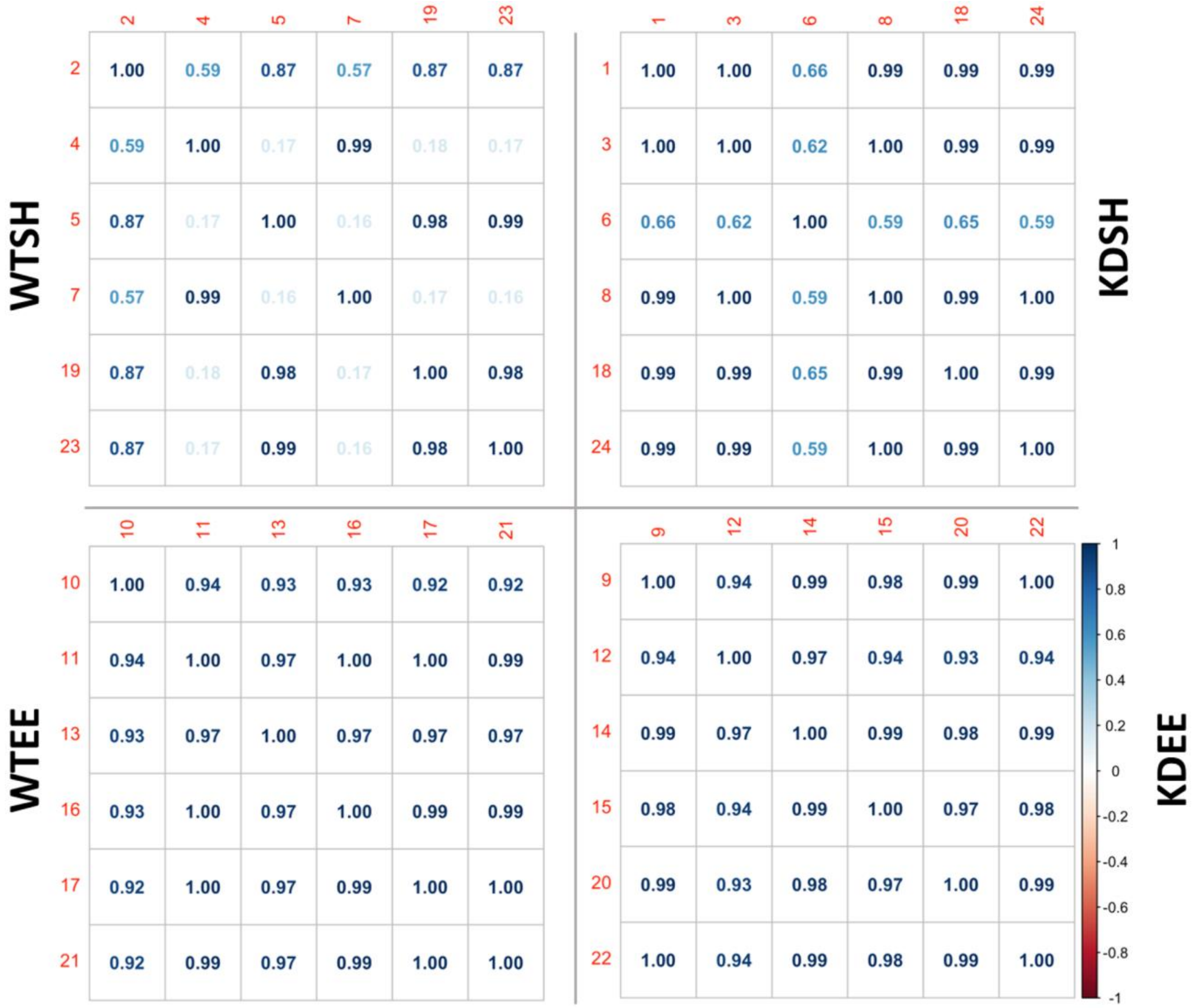

Supplementary Figure 6. Intragroup correlation matrices comparing Pearson's correlation coefficient values between samples from same group. Note poor correlation ($r < 0.9$) in samples 4, 7 (WTSH) and 6 (KDSH).

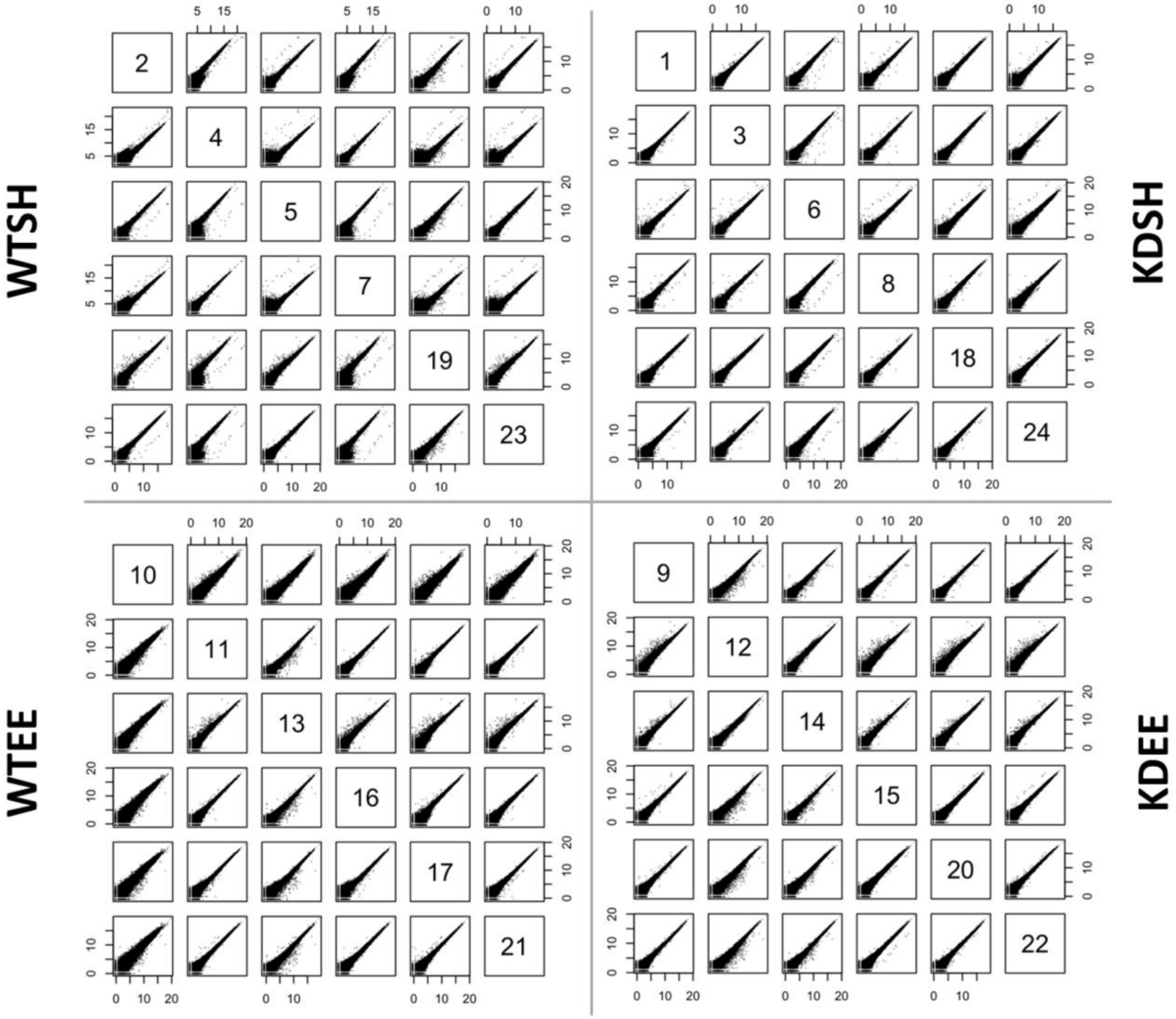

Supplementary Figure 7. Intragroup gene count correlation plots. Note poor correlation in samples 4, 7 (WTSH) and 6 (KDSH). Samples 4 and 7 consistently correlate together. This can also be observed in the Pearson's plots (Supplementary Figure 6). Sample 10 exhibits greater variation compared to other samples in group, but Pearson's r still > 0.9.
