# Supplementary material for: MSK1 is required for the beneficial synaptic and cognitive effects of enriched experience across the lifespan
Source: Aging (Albany NY). 2023 Jul 10;15(13):6031–72. doi: 10.18632/aging.204833 (PMC10373962; doi:10.18632/aging.204833)
Supplement: Supplementary Tables [file aging-15-204833-s002.pdf]

SUPPLEMENTARY TABLES

Supplementary Table 1. Sample data for all samples.

Sample name	File name	Condition	Genotype	Housing
1	Sample_1.txt	KDSH	mut	SH
3	Sample_3.txt	KDSH	mut	SH
6	Sample_6.txt	KDSH	mut	SH
8	Sample_8.txt	KDSH	mut	SH
18	Sample_18.txt	KDSH	mut	SH
24	Sample_24.txt	KDSH	mut	SH
9	Sample_9.txt	KDEE	mut	EE
12	Sample_12.txt	KDEE	mut	EE
14	Sample_14.txt	KDEE	mut	EE
15	Sample_15.txt	KDEE	mut	EE
20	Sample_20.txt	KDEE	mut	EE
22	Sample_22.txt	KDEE	mut	EE
2	Sample_2.txt	WTSH	WT	SH
4	Sample_4.txt	WTSH	WT	SH
5	Sample_5.txt	WTSH	WT	SH
7	Sample_7.txt	WTSH	WT	SH
19	Sample_19.txt	WTSH	WT	SH
23	Sample_23.txt	WTSH	WT	SH
10	Sample_10.txt	WTEE	WT	EE
11	Sample_11.txt	WTEE	WT	EE
13	Sample_13.txt	WTEE	WT	EE
16	Sample_16.txt	WTEE	WT	EE
17	Sample_17.txt	WTEE	WT	EE
21	Sample_21.txt	WTEE	WT	EE

Table includes those samples subsequently failing quality control (QC) and not taken forward for analysis. Abbreviations: WT: Wild-type; KD: MSK1 Kinase Dead (KD); mut: mutant; SH: standard-housed; EE: environmental enrichment.

Supplementary Table 2. Sample data for samples used in RNAseq DEG analysis.

Sample name	File name	Condition	Genotype	Housing
1	Sample_1.txt	KDSH	mut	SH
3	Sample_3.txt	KDSH	mut	SH
8	Sample_8.txt	KDSH	mut	SH
18	Sample_18.txt	KDSH	mut	SH
24	Sample_24.txt	KDSH	mut	SH
9	Sample_9.txt	KDEE	mut	EE
12	Sample_12.txt	KDEE	mut	EE
14	Sample_14.txt	KDEE	mut	EE
15	Sample_15.txt	KDEE	mut	EE
20	Sample_20.txt	KDEE	mut	EE
22	Sample_22.txt	KDEE	mut	EE
2	Sample_2.txt	WTSH	WT	SH
5	Sample_5.txt	WTSH	WT	SH
19	Sample_19.txt	WTSH	WT	SH

23	Sample_23.txt	WTSH	WT	SH
10	Sample_10.txt	WTEE	WT	EE
11	Sample_11.txt	WTEE	WT	EE
13	Sample_13.txt	WTEE	WT	EE
16	Sample_16.txt	WTEE	WT	EE
17	Sample_17.txt	WTEE	WT	EE
21	Sample_21.txt	WTEE	WT	EE

Samples 4, 6 and 7 have been excluded due to failing QC. Abbreviations as per Supplementary Table 1.
